# Site-Specific Abiraterone Protein–Drug Conjugates via Hedgehog Autoprocessing

**DOI:** 10.1021/acsami.5c22490

**Published:** 2026-04-09

**Authors:** Asma Gulzar, Laiba Maryam, Sisila Valappil, Vivanthi Thanthrige, Ebbing de Jong, Miguel Guzman, Md Shahadat Hossain, Atanu Acharya, Davoud Mozhdehi

**Affiliations:** † Department of Chemistry, 2029 Syracuse University, 111 College Place, Syracuse, New York 13244, United States; ‡ Upstate Medical University, Proteomics and Mass Spectrometry, Weiskotten Hall 4307 WHA, 766 Irving Avenue, Syracuse, New York 13210, United States; § BioInspired Syracuse: Institute for Material and Living Systems, Syracuse University, Syracuse, New York 13244, United States

**Keywords:** lipoengineering, abiraterone, hedgehog, post-translational modification, protein–drug conjugate, prostate cancer

## Abstract

Protein lipoengineering is a promising approach for programming biomolecular assembly and function, yet its use has remained confined to biologically inert lipids. Here, we repurpose the hedgehog autoprocessing domain as a biocatalyst to install the anticancer sterol abiraterone as a noncanonical post-translational modification (ncPTM) on an intrinsically disordered protein scaffold. Artificial abirateronylation produced protein–drug conjugates (PDCs) with significantly enhanced solubility, tunable phase behavior, and dynamic self-association. The conjugates underwent slow hydrolytic release of abiraterone and reproduced the cytotoxic response of the free drug in prostate-cancer cells, achieving more uniform activity and distributions in 3D tumor spheroids. By extending this approach to a second steroidal prostate-cancer agent, galeterone, we demonstrate a generalizable ncPTM strategy for exploiting native lipidation machinery to construct molecularly defined, programmable protein–drug therapeutics.

## Graphical abstract

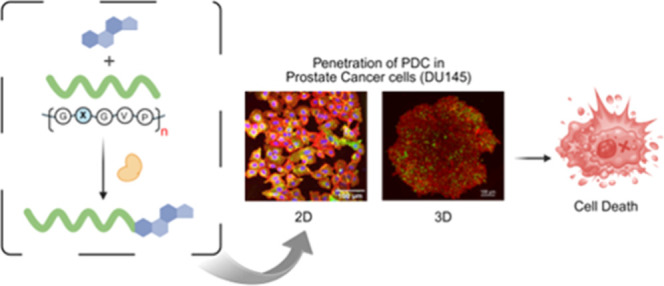

## Introduction

Post-translational modifications (PTMs) offer a vast yet underexploited design space for engineering protein therapeutics and biomaterials. Inspired by the successes of glyco and phosphoengineering in improving the stability and efficacy of biologics,^1–6^ lipoengineering is emerging as a powerful molecular design strategy for programming the assembly and function of proteins.^7^ By precisely controlling the amphiphilic architecture of the hybrid biopolymer, the conjugation of lipids such as fatty acids,^8–11^ sterols,^12–14^ farnesyl,^15,16^ or geranylgeranyl^17^ groups can regulate the nano- and mesoscale organization of hybrid nanobiomaterials, including stimuli-responsive nanoparticles for drug delivery and multivalent scaffolds for displaying bioactive peptides.^18–22^ Despite these advancements, most lipoengineered systems so far have relied on biologically inert lipids that serve purely structural roles, overlooking the vast functional diversity of bioactive lipids that act as signaling molecules (e.g., eicosanoids),^23,24^ immunomodulators (e.g., *N*-acyl-*S*-diacylglycerols),^25,26^ and pharmacological agents (e.g., steroids).^27^ Expanding lipoengineering to encompass such bioactive chemistries could transform it from a structural design tool into a route for generating functional therapeutics.

However, site-specific conjugation of pharmacologically active lipids presents both conceptual and practical challenges owing to their extreme hydrophobicity and lack of bioorthogonal functional groups.^28,29^ Despite substantial advances in protein modification methods,^30^ including native or bioorthogonal handles (e.g., cysteine–maleimide^31,32^ or alkyne–azide click reaction^33,34^ ), coupling hydrophobic payloads to proteins under mild, biocompatible conditions remains difficult and often requires labor-intensive optimization of organic cosolvents or surfactants to maintain payload solubility on a case-by-case basis.^35,36^ Biocatalytic strategies offer a promising alternative: enzymes that natively mediate lipid transfer frequently exhibit remarkable substrate plasticity, enabling the accommodation of diverse hydrophobic scaffolds in aqueous environments.^37^ Although this substrate plasticity has been exploited for profiling lipidated proteins,^38^ its potential in biomaterials engineering remains untapped.^34,39^

Hedgehog proteins provide a compelling precedent for this concept. Their biological activity depends on a unique post-translational cholesterylation of their signaling domain, catalyzed by an autoprocessing C-terminal domain. This autoprocessing domain functions as a “gated” intein, where cholesterol binding to a sterol recognition motif triggers the formation of a thioester bond at the junction of two domains, which is subsequently reacted with sterol.^40^ Previous work has established that this C-terminal domain can be repurposed as a biotechnology tool to ligate cholesterol or sterol analogs bearing biorthogonal handles onto non-native protein partners, generating self-assembling hybrid nanomaterials (Figure S4).^13,14,41,42^

Inspired by these precedents, we sought to extend the utility of this autoprocessing domain as a biocatalyst for site-specific lipidation of proteins with pharmacologically active sterols as a noncanonical PTM (ncPTM), Figure 1. Specifically, we asked whether it could conjugate abiraterone—a frontline steroidal anticancer drug plagued by extremely low aqueous solubility (<0.1 μM) and food-dependent bioavailability^43^—to a recombinant carrier to produce protein–drug conjugates (PDCs) with tunable pharmacological properties. We show that the wildtype *Drosophila melanogaster* hedgehog abirateronylates a library of elastin-derived intrinsically disordered protein polymers (IDPP) to yield molecularly defined PDCs with cleavable ester linkages (Table S1). By optimizing the carrier protein, we identify a construct with over 2000-fold greater aqueous solubility compared to free abiraterone that exhibits superior cytotoxicity in 3D prostate cancer spheroids. Our study establishes a proof of concept that this ncPTM strategy can incorporate bioactive lipids into recombinant proteins with molecular precision, enabling modular and programmable platforms for therapeutic design.

**Figure 1:**
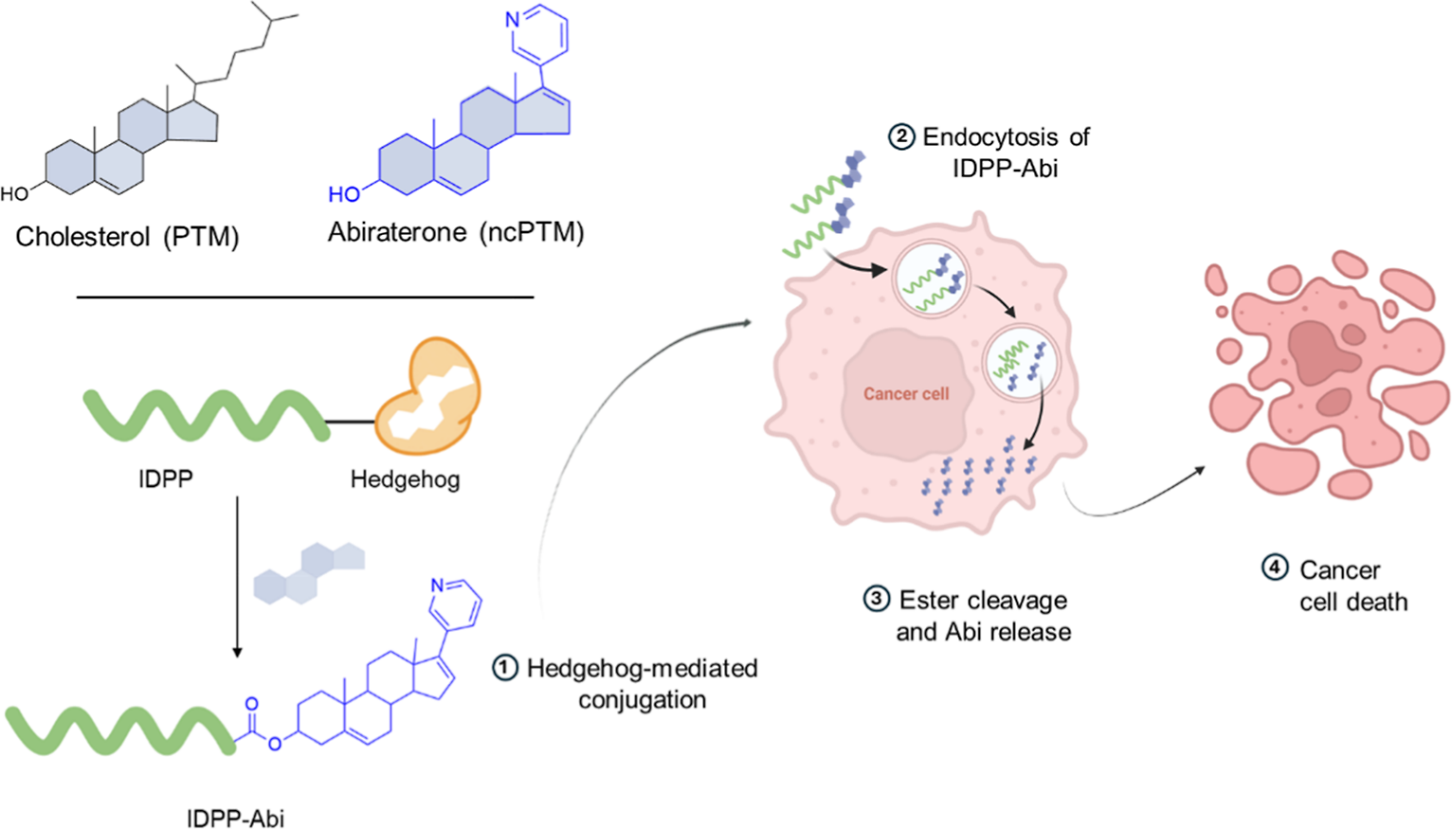
Schematic of hedgehog-mediated abirateronylation as a noncanonical PTM (ncPTM) for generating protein–drug conjugates (PDCs). An intrinsically disordered protein polymer (IDPP) serves as the model protein scaffold. The resulting hybrid biopolymers are internalized by DU145 cells and exert cytotoxicity. Created with BioRender.com

## Results and Discussion

To evaluate whether sterol structure influences recognition by the hedgehog autoprocessing domain, we analyzed the *D. melanogaster* hedgehog C-terminal domain—the biocatalyst for abirateronylation—using molecular dynamics and ligand docking (Figures 2, S1–S3). Compared to its native substrate, cholesterol (Chol), Abi replaces the flexible alkyl substituent at ring D with a rigid pyridine moiety, a modification that could perturb recognition within the sterol recognition motif (SRM) and subsequent catalytic activation. The simulations revealed that the partially ordered SRM has sufficient plasticity (Figure 2a) to accommodate abiraterone (Abi) in a catalytic geometry convergent with its native substrate, cholesterol (Chol). Both sterols adopted similar poses at the interface of intein and SRM domains (Figures 2b and S3), placing the reactive hydroxyl near the catalytic cysteine (C1) and the activating residue D46, consistent with prior mechanistic studies.^40,44^ Differences were most apparent at the sterol tail, with Chol’s aliphatic side chain preferentially interacting with hydrophobic A71 and V146, whereas Abi’s pyridine interacts with D90.

**Figure 2:**
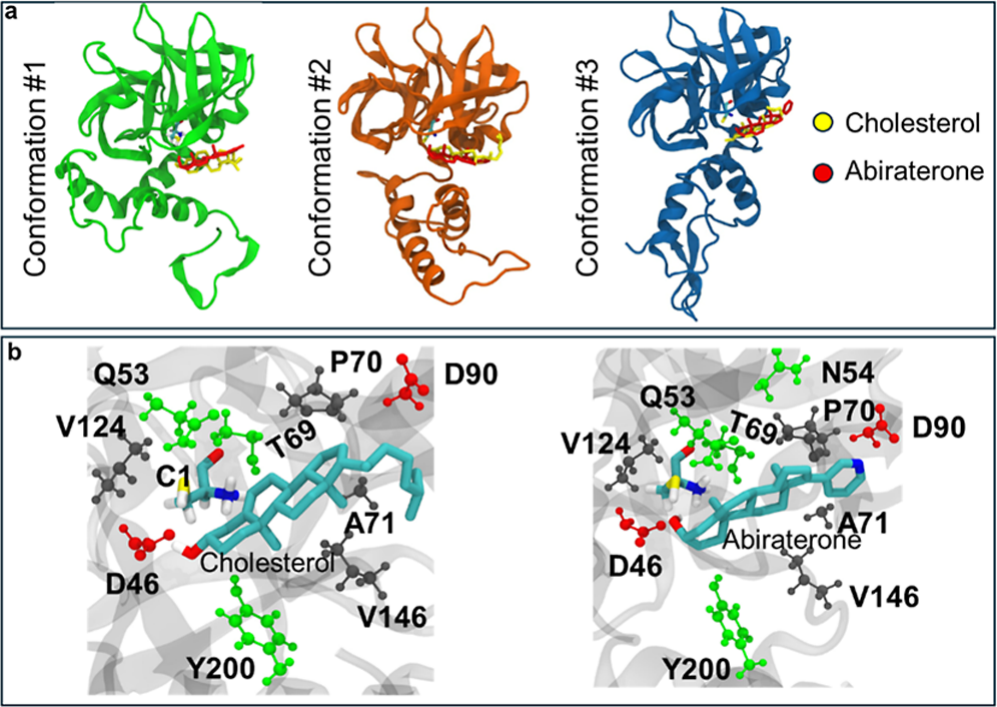
Substrate plasticity of Hedgehog C-terminal domain. (a) Representative structures of conformational clusters of the Hedgehog protein and docked substrates. Chol and Abi were docked to three conformations obtained from clustering of 400 ns MD simulations at a similar docking site. (b) Interaction between docked Chol and Abi in conformation 1. Both molecules adopt a conserved orientation of their hydroxyl groups and interact with similar residues. See Figure S3 for representative docked structures with conformations 2 and 3.

Consistent with these in-silico predictions, Abi proved to be an efficient substrate for *D. melanogaster* hedgehog autoprocessing domain, activating the N → S acyl shift, self-cleavage, and sterol-modification with efficiency comparable to Chol. When a fusion construct composed of an elastin-like polypeptide (E) and the Hedgehog C-terminal domain (H) was expressed in *E. coli* and incubated with either sterol in lysate, the full-length EH precursor (46 kDa) was consumed, and a new band corresponding to the cleaved autoprocessing domain appeared at 25 kDa (Figure 3a). Densitometric analysis conducted on stain-free gels showed a comparable extent of autoprocessing for either substrate (82 ± 3% for Chol and 77 ± 4% for Abi) within 3 h, whereas the no-sterol control exhibited only baseline hydrolysis (15 ± 2%). Because differences in binding mode of Abi or Chol to the SRM could alter catalytic geometry, we next determined whether activation proceeded via productive sterolysis or off-pathway thioester hydrolysis (Figure S4)—a critical distinction given the irreversible, single-turnover nature of Hedgehog autoprocessing. Product distributions were analyzed by RP-HPLC, exploiting the increased hydrophobicity and retention time of sterol-modified conjugates (E–Abi or E–Chol) relative to the hydrolyzed carrier (E–OH). Precursor consumption correlated linearly with ligated product formation for both sterols (Figure 3b), indicating tight coupling between autoprocessing and conjugation. Both reactions yielded high modification efficiencies (82.6 ± 2.5% for E–Abi and 83 ± 5.2% for E–Chol), whereas no-sterol controls or β-mercaptoethanol (a competing thiol nucleophile) only produced background hydrolysis. The modest increase in precursor consumption observed in the presence of β-mercaptoethanol (49 ± 3%) is consistent with the notion that sterol binding to SRM is required to promote the formation of reactive thioester intermediate. While Triton X-100 was included in the lysate to ensure sterol solubilization, its presence did not significantly impact autoprocessing or modification efficiencies (Figure S5), indicating that the EH fusion can likely solubilize sterols even in the absence of surfactants.

**Figure 3:**
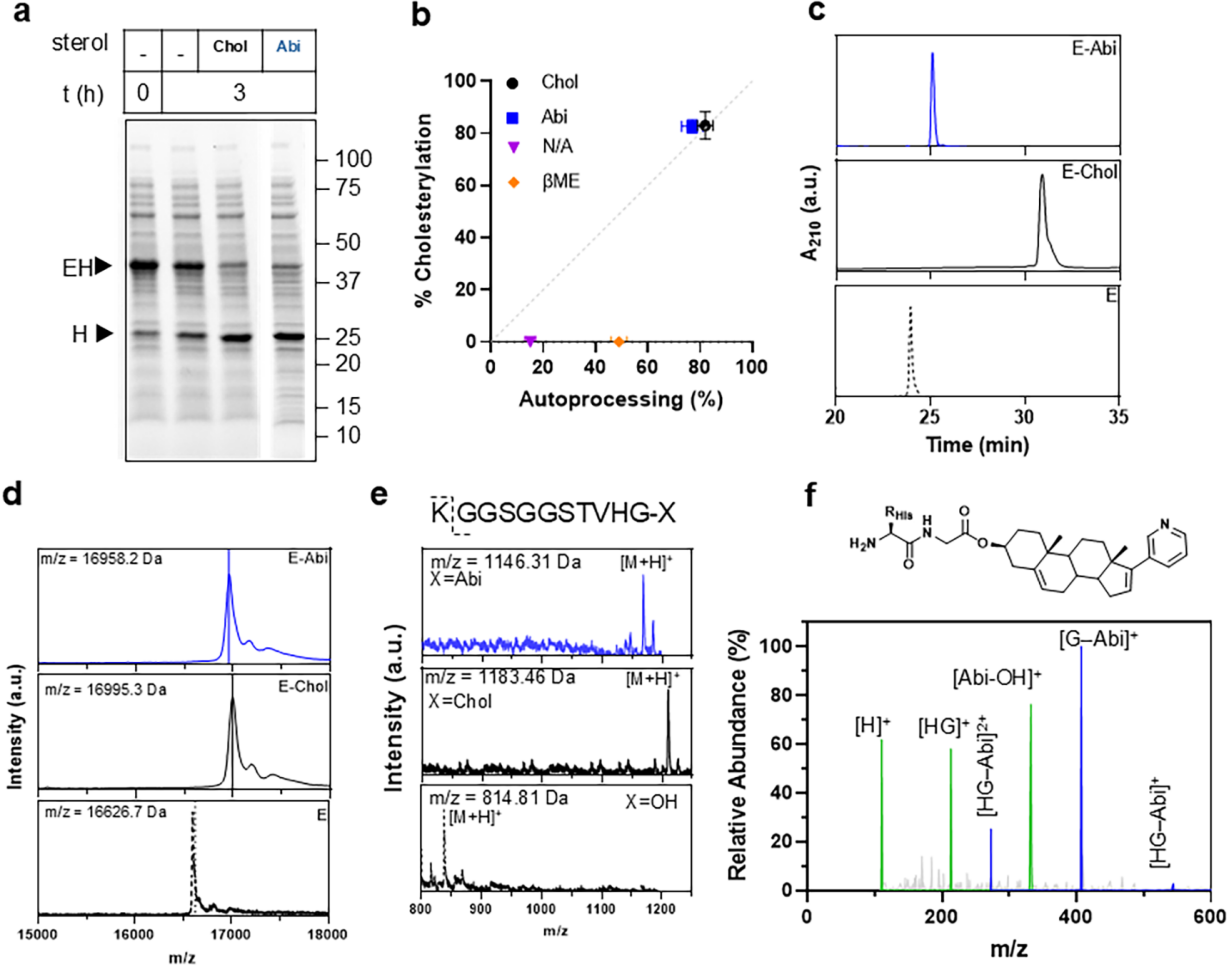
Efficient and site-specific biocatalytic conjugation of abiraterone (Abi) to proteins. (a) Representative SDS-PAGE gel of the autoprocessing reaction after 3 h. Both native (Chol) and noncanonical substrates (Abi) trigger the consumption of full-length protein precursor (EH), and the appearance of the cleaved H-domain. (b) Correlation between precursor consumption (from densitometry) and sterolylation efficiency (from HPLC). (c) RP-HPLC traces of purified E–OH, E–Abi, and E–Chol. The increase in retention time correlates with C-termini hydrophobicity. (d) MALDI-TOF mass spectrometry E, E-Abi, and E-Chol confirming the addition of a single abiraterone (+331.5 Da) or cholesterol (+368.6 Da) moiety. (e) MALDI-TOF analysis of tryptic digests localizes the mass shift to the C-terminal peptide fragment. (f) Tandem LC–MS/MS spectrum of the C-terminal fragments from E–Abi. Data in b are mean ± s.d. (*n* = 3).

Finally, to definitively confirm the identity and site-specificity of abirateronylation, we isolated the products (Figure S6) and characterized the purified proteins with RP-HPLC and mass spectrometry (Figure 3c–f). RP-HPLC showed that the retention times of biopolymers correlated with the hydrophobicity of their C-termini (Figure 3c): Unmodified carrier, bearing a C-termini carboxylic acid eluted first (23.9 min), followed by E–Abi (25.0 min) on a C18 column, whereas E–Chol required a less retentive C4 column, where it still eluted at 30.9 min, consistent with its substantially higher hydrophobicity. MALDI-TOF mass spectrometry confirmed the covalent addition of a single sterol moiety and release of a water molecule, for both E–Abi (+331.5 Da) and E–Chol (+368.6 Da) (Figure 3d). To pinpoint the modification site, trypsin-digested samples were analyzed by MALDI-TOF (Figure 3e, Table S3) and LC–MS (Figure S7), which localized the mass shift to the C-terminal peptide fragment (GGSGGSTVHG-X). This was conclusively confirmed by tandem LC–MS/MS analysis (Figure 3f), which identified the fragment ion series mapping the abiraterone modification to the C-terminal glycine residue (Table S4). Together, these data conclusively establish that the hedgehog autoprocessing domain can be repurposed as a “drug ligase” to site-specifically conjugate abiraterone, a non-native therapeutic sterol, onto a protein carrier with high efficiency and precision.

Having established the feasibility of this biocatalytic strategy, we next asked whether it could address one of abiraterone’s key pharmacological limitations—its extremely low aqueous solubility, which leads to inconsistent oral bioavailability and large variability in patient exposure depending on food intake.^43^ While PEGylation is a validated strategy to improve conjugate solubility,^45,46^ we hypothesized that a thermoresponsive protein carrier could similarly achieve enhanced solubility while enabling programmable control over the conjugate’s physicochemical behavior. As a model carrier, we selected IDPPs based on elastin-like polypeptides (ELPs), which undergo reversible, temperature-dependent liquid–liquid phase separation (LLPS).^47–49^ Because their LLPS can be tuned by adjusting their composition (e.g., by varying the hydrophobicity of the guest residue or biopolymer length),^50^ we envisioned that abirateronylation of these biopolymers would yield modular hybrids in which sequence changes within the E-domain enable control over pharmacologically relevant behaviors such as colloidal stability, nanoparticle formation, and thermally triggered depot formation—features that are difficult to encode at the small-molecule level.

To establish a predictive framework for conjugate design and identify a construct for biological studies, we characterized the temperature-dependent phase transition for a library of (GXGVP)_*n*_ scaffolds (Table S1 and Figure S8), varying both the guest residue (X) and chain length (*n*). Sterol-conjugates preserved the reversible LLPS behavior of the parent ELPs but exhibited markedly reduced transition temperatures (*T*_t_). For example, modifying a (GVGVP)_30_ carrier reduced its *T*_t_ from 40.5 to 28 °C (E–Abi), and 27 °C (E–Chol) (Figure 4a). The nearly identical *T*_t_ values of E–Chol and E–Abi—despite differences in the sterol hydropathy—indicate that the thermoresponsive behavior of the conjugate is controlled by a nonadditive interplay between the hydrophobicity of the lipid and ELP composition. To decouple these variables, we fit the *T*_t_ of the unmodified ELP (E) and corresponding E–Abi to *T*_t_ = *T*_c_ + *m* × ln­(*C*), where *T*_c_ is the critical transition temperature at dilute condition (i.e., 1 μM), *C* is protein concentration (1–200 μM in PBS), and *m* quantifies the concentration dependence of *T*_t_, Figures 4b and S9. This analysis revealed that abiraterone modulates carrier phase behavior through two distinct mechanisms. First, end-group lipidation drives sequence-dependent changes in chain hydration that depresses *T*_c_ at low concentrations (Δ*T*_c_ = −12 °C to −55 °C), with a poor correlation between the unmodified ELP’s *T*_c_ and Δ*T*_c_. Second, at higher concentrations, the Abi conjugates undergo concentration-dependent association, evidenced by a flattened concentration dependence (reduced m), consistent with dynamic oligomerization above an apparent critical micellar concentration (CMC), 60 μM for (V,30), 100 μM for (A,40), and 150 μM for (V8/A2/K,80) (Figure 4b, arrows). Guided by these biophysical insights, we selected the (A,40) construct as the optimal candidate for further development, owing to its favorable physicochemical profile. Even at concentrations exceeding 200 μM—representing more than a 2000-fold increase in solubility relative to free abiraterone—the E–Abi­(A,40) conjugate remained colloidally stable across physiologically relevant temperatures (37–42 °C). Although we focus here on solubility, this framework should enable tuning of conjugate pharmacokinetics by programming thermally triggered drug depots with distinct dissolution kinetics in future studies.^51^

**Figure 4:**
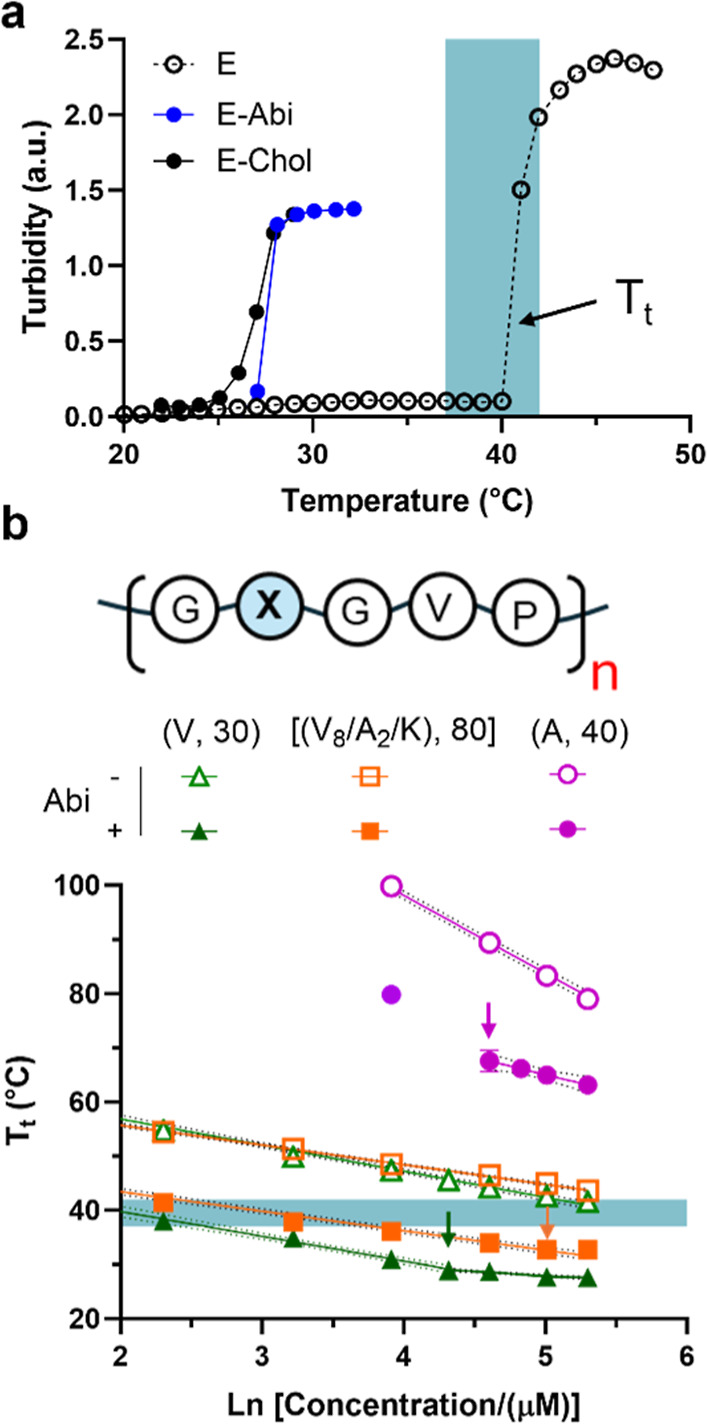
Composition-dependent effects of abirateronylation on biopolymer phase behavior. (a) Representative variable-temperature turbidity profiles for (GVGVP)_30_ biopolymer and its conjugates at 50 μM in PBS. Both E–Chol and E–Abi exhibit significantly lower transition temperature (*T*_t_) compared to the unmodified (E) carrier, showing that sterol conjugation lowers *T*_t_ via nonadditive hydrophobic effects. (b) Temperature–concentration phase diagrams for (GXGVP)_*n*_ scaffolds, native (open symbols) vs abiraterone-modified (filled symbols), highlighting composition-dependent shifts in intercept and slope. Arrows indicate onset of nonlinearity consistent with oligomer formation above the critical micellar concentration. The blue shaded region represents the physiologically relevant temperature range (37–42 °C). Data are mean ± s.d. (*n* = 3). Dotted lines indicate the 95% confidence interval of the linear fit. *T*_t_ for the (A,40) variant exceeded the measurable range and was extrapolated using an empirical model.^50^

Consistent with the turbidimetry results, DLS revealed that E–Abi­(A,40) undergoes concentration-dependent assembly (Figures 5a and S10). At 200 μM, E–Abi formed dynamic oligomers (*R*_h_ = 17.5 ± 2.7 nm) that partially dissociated upon dilution to 50 μM (*R*_h_ = 12.9 ± 0.7 nm). This behavior contrasted with the unmodified carrier, which remained unimeric (*R*_h_ = 7.2 ± 0.3 nm), and E–Chol, which formed large nanoparticles (*R*_h_ = 50 ± 0.5 nm) at both concentrations. SEC-MALS further supported the dynamic nature of E–Abi assemblies (Figure S11), as E–Abi eluted as a monomeric peak, consistent with rapid disassembly under column dilution, whereas E–Chol retained higher-order species, consistent with its stronger and less reversible associations. Collectively, these data indicate that abirateronylation promotes weak, reversible self-association, which should be factored into carrier design to maintain colloidal stability at high concentrations.

**Figure 5:**
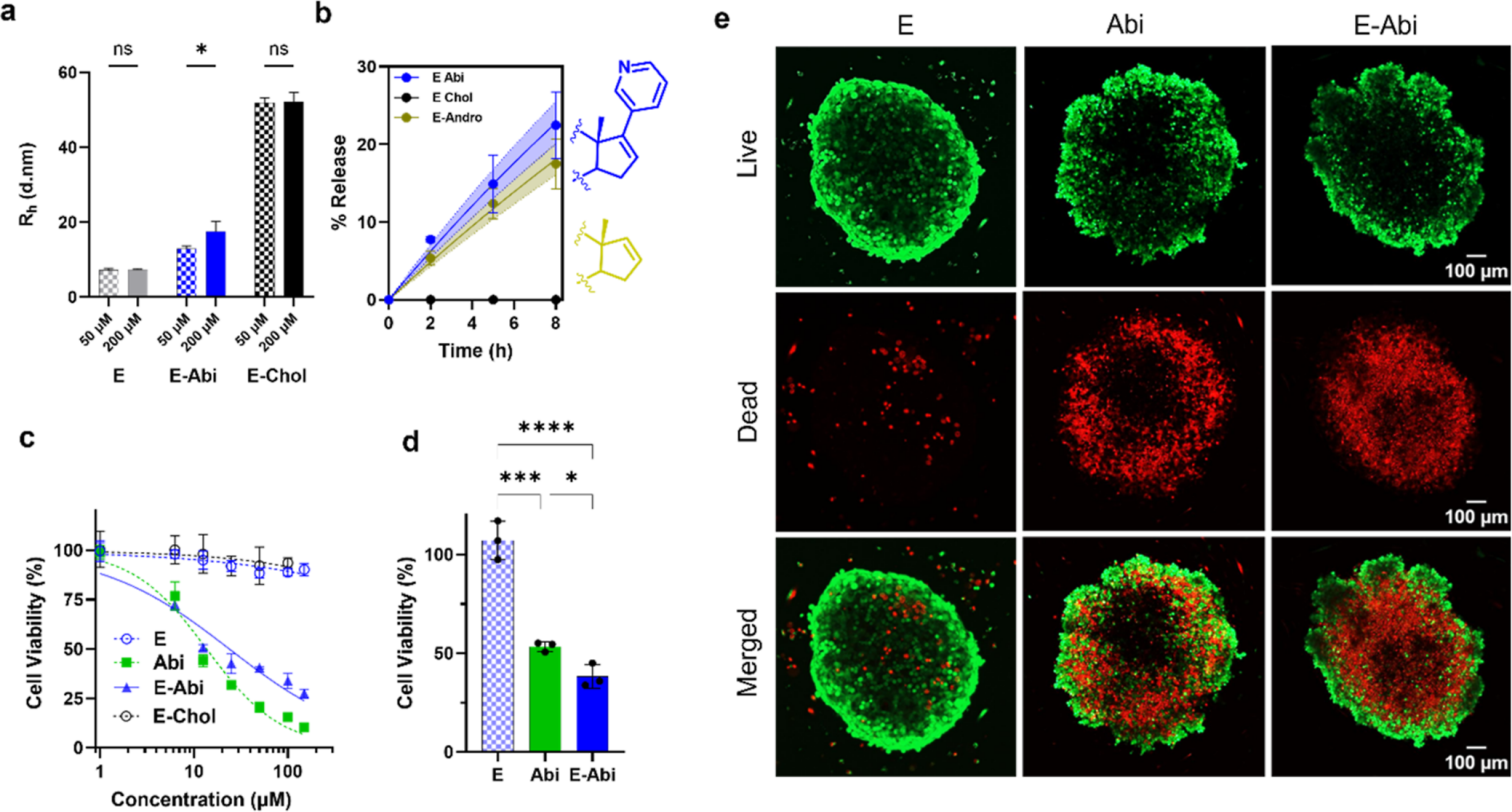
Biophysical and biological assessment of the optimized PDC and controls. (a) Hydrodynamic radius (*R*_h_) of (A,40) carrier (E), E–Abi and E–Chol at 50 μM and 200 μM, measured by dynamic light scattering. E–Abi forms reversible, concentration-dependent oligomers. (b) Release kinetics of conjugated sterols (E-Abi, E–Andro and E–Chol) in PBS (pH 6.5) at 37 °C, along with the chemical structures of Abi and Andro tail groups. E–Abi and E–Andro shows sustained release, whereas E–Chol displays negligible sterol release over the same period. Shaded areas represent the 95% confidence intervals of the nonlinear regression fit to a first-order kinetics model. (c) Dose–response curves for DU145 prostate cancer cells after 72 h exposure to free drug, conjugate, and controls (measured by MTT assay; nonlinear dose–response fit). (d) Viability of 3D DU145 spheroids after 24-h treatment with 100 μM of each compound, showing superior efficiency of E–Abi. (e) Representative live/dead fluorescence images of treated spheroids. Live cells are stained green (calcein AM), and dead cells are stained red (propidium iodide). Data are mean ± s.d. (*n* = 3). Statistical analysis was performed using one-way ANOVA with posthoc tests (Tukey’s for a, Holm–Sidak’s for d): *p < 0.05, **p < 0.01, ***p < 0.001 and ****p < 0.0001.

Because abiraterone must be liberated from the carrier to exert its pharmacological activity, we evaluated the hydrolytic stability of the E–Abi conjugate in buffer and serum. In PBS, E–Abi exhibited slow, controlled release, reaching approximately 30% after 8 days, whereas E–Chol showed negligible hydrolysis (Figure 5b). MALDI-TOF analysis confirmed that the cleavage occurred via ester hydrolysis and not backbone degradation (Figure S13). Initially, we hypothesized that the pyridine moiety of abiraterone might promote hydrolysis via nucleophilic catalysis. However, evaluation of an androstenol conjugate (E–Andro)—a sterol lacking the pyridine substituent—argued against this mechanism, as E–Andro displayed a comparable hydrolysis rate (*t*_1/2_ = 21 days, 95% CI [18.8, 24.8]) to E-Abi. Instead, the inverse correlation between initial release kinetics and construct hydrophobicity (E–Abi < E–Andro ≪ E–Chol; Figure S14), consistent with an assembly mediated shielding model in which more hydrophobic, colloidally stable particles exclude water more effectively near the ester linkage. Direct measurements of local hydration (e.g., using ODNP^52^) will be valuable to test this hypothesis. While hydrolysis in pure buffer was slow, we anticipated faster cleavage in biological environments abundant in esterases. Accordingly, increasing the fetal bovine serum (FBS) content accelerated the hydrolysis rate, decreasing the projected half-life of the conjugate to 6.7 days (10% FBS), and 4.5 days (50% FBS), Figure S15. Crucially, despite its hydrolytic susceptibility, the conjugate remained stable for weeks during storage as either a lyophilized powder or a frozen solution (Figure S12). Collectively, these results demonstrate that E–Abi functions as a colloidally stable prodrug and the observed biological activity (discussed below) derives from the delivered payload.

For this prodrug strategy to be effective, cellular internalization must occur on a time scale faster than extracellular hydrolysis.^53^ We therefore quantified uptake of fluorescently labeled carriers in DU145 prostate cancer cells after a 3 h incubation—a window in which drug release is minimal—using confocal microscopy and flow cytometry. Both the unmodified carrier (E) and E–Abi showed robust internalization, displaying punctate intracellular fluorescence and a high fraction of uptake-positive cells (61 ± 4.7% and 63 ± 4.5%, respectively; Figure S16), whereas the more hydrophobic E–Chol exhibited predominantly peripheral fluorescence and markedly reduced internalization (14.8 ± 0.4%), consistent with membrane adsorption rather than productive cellular uptake (Figure S16). Pharmacological inhibition further indicated that E–Abi internalization proceeds primarily via dynamin-dependent, caveolae-mediated endocytosis (dynasore and genistein), with minimal contribution from actin-dependent pathways such as macropinocytosis (cytochalasin D), Figure S17.^54^ Together, these results support that most of the conjugate is internalized in its sterol-modified state, given the temporal disparity between fast uptake kinetics and much slower hydrolytic release. We are cognizant, that because E–Abi oligomerizes reversibly rather than forming stable nanoparticles, these results are best interpreted as reflecting endocytic internalization of a dynamic ensemble of sterol-modified species.

Encouraged by these results, we compared the antiproliferative activity of E–Abi in 2D monolayer culture of DU145 prostate cancer cells relative to controls. Cell viability was quantified over 96 h by MTT assay after treatment with unmodified carrier (E), free abiraterone (Abi), E–Abi, and E–Chol across a 1–150 μM dose range (Figures 5c and S18 for 95% confidence intervals). Whereas E and E-Chol showed minimal cytotoxicity, Abi and E–Abi elicited dose-dependent reductions in viability with distinct temporal profiles. Free Abi induced rapid cytotoxicity (IC_50_ = 15 μM at 24 h), whereas E–Abi displayed a delayed onset, with viability reduced by 35 ± 1.4% (24 h) and 51 ± 4.2% at 48 h at the highest dose (Figure S18a,b). With longer incubation, E–Abi efficacy progressively improved, matching free Abi by 72 h (Figure 5c) and exceeding it slightly by 96 h (IC_50_ = 10 μM vs 17 μM for Abi; Figure S18d), a trend consistent with sustained intracellular exposure from PDCs.^55^ LC–MS analysis of DU145 lysates confirmed that intracellular Abi concentration increases over time following E–Abi treatment (Figure S19), reaching comparable levels to free drug administration within 96 h. This gradual accumulation, which is consistent with the hydrolysis rate in biological media (Figure S15) and the observed lag in cytotoxicity, confirms that E–Abi function as a macromolecular prodrug conjugate. Live/dead fluorescence imaging further corroborated these findings (Figure S20), and control experiments showed that E–Abi exhibited minimal toxicity in NIH-3T3 fibroblasts after 24 h exposure (Figure S21), defining an early tolerability window, consistent with the higher homeostatic threshold of nonmalignant cells compared to cancer phenotypes.^56,57^ Overall, these results indicate that the characteristic prodrug lag of E–Abi occurs without compromising efficacy. Furthermore, the protein carrier confers enhanced solubility and colloidal stability, features expected to be particularly advantageous in more physiologically complex environments.

To evaluate these advantages under conditions that better emulate the tumor microenvironment, we next assessed the cytotoxic performance of E–Abi in three-dimensional (3D) DU145 spheroids, which impose additional constraints such as limited diffusion and spatial heterogeneity in cell accessibility. E–Abi exhibited superior cytotoxic activity compared to free abiraterone in this model at 100 μM (Figure 5d), whereas at 150 μM both treatments produced comparable levels of growth inhibition (Figure S22). Moreover, live/dead confocal imaging (Figure 5e) showed that spheroids treated with free Abi exhibited localized cell death primarily in the periphery of the spheroids, whereas those treated with E–Abi showed more uniform cell death throughout the spheroid.

To connect the observed cytotoxicity patterns with intratumoral distribution, we examined the uptake and penetration of fluorescently labeled carriers in DU145 spheroids by flow cytometry and confocal microscopy following 24 h exposure (Figure 6). In this 3D context, the hydrophobicity of the biopolymer’s C-terminus strongly influenced both internalization and spatial localization. Following spheroid dissociation and trypan blue quenching, flow cytometry revealed that only 3.1 ± 1.8% of cells internalized unmodified biopolymer (E), whereas E–Abi and E–Chol achieved significantly higher uptake (20.9 ± 4.5% and 23.3 ± 4.8%, respectively; Figure 6a–d). The difference from the monolayer uptake assay (Figure S16) likely arises from differences in fluid-phase entry in the dense spheroid matrix, coupled with the longer exposure window (24 h vs 3 h). Confocal microscopy corroborated these trends: unmodified E showed negligible signal within spheroids (Figure 6e), whereas E–Abi and E–Chol produced strong fluorescence within spheroids but with distinct spatial distributions (Figure 6f,g). Radial segmentation of z-stacks showed that E–Abi penetrated broadly across the spheroid depth, reaching ∼360 μm, whereas E–Chol accumulated predominantly in the peripheral shell (∼120 μm), forming a steeper gradient that decayed toward the core (Figure S23). Because both proteins remained soluble under these conditions (Figure S10), the observed penetration profiles primarily reflect and microenvironment-mediated transport limitation (e.g., binding site barriers) rather than phase-driven sequestration.^58–63^ This trend parallels observations for lipidated short peptides (e.g., GLP-1R agonists), where increasing lipid hydrophobicity enhances membrane association but may alter tissue penetration.^64^ The deeper diffusion of E–Abi compared to its more hydrophobic E–Chol isoform suggests that a similar principle may apply to the localization and distribution of larger proteins—with an optimal “sweet spot” of hydrophobicity for achieving the desired biodistribution. Collectively, these findings suggest that conjugation of abiraterone to a soluble carrier protein may broaden its therapeutic index and facilitate uniform intratumoral delivery, by reducing the concentration gradients that limit the efficacy of hydrophobic therapeutics in dense tumor environments.^54^

**Figure 6:**
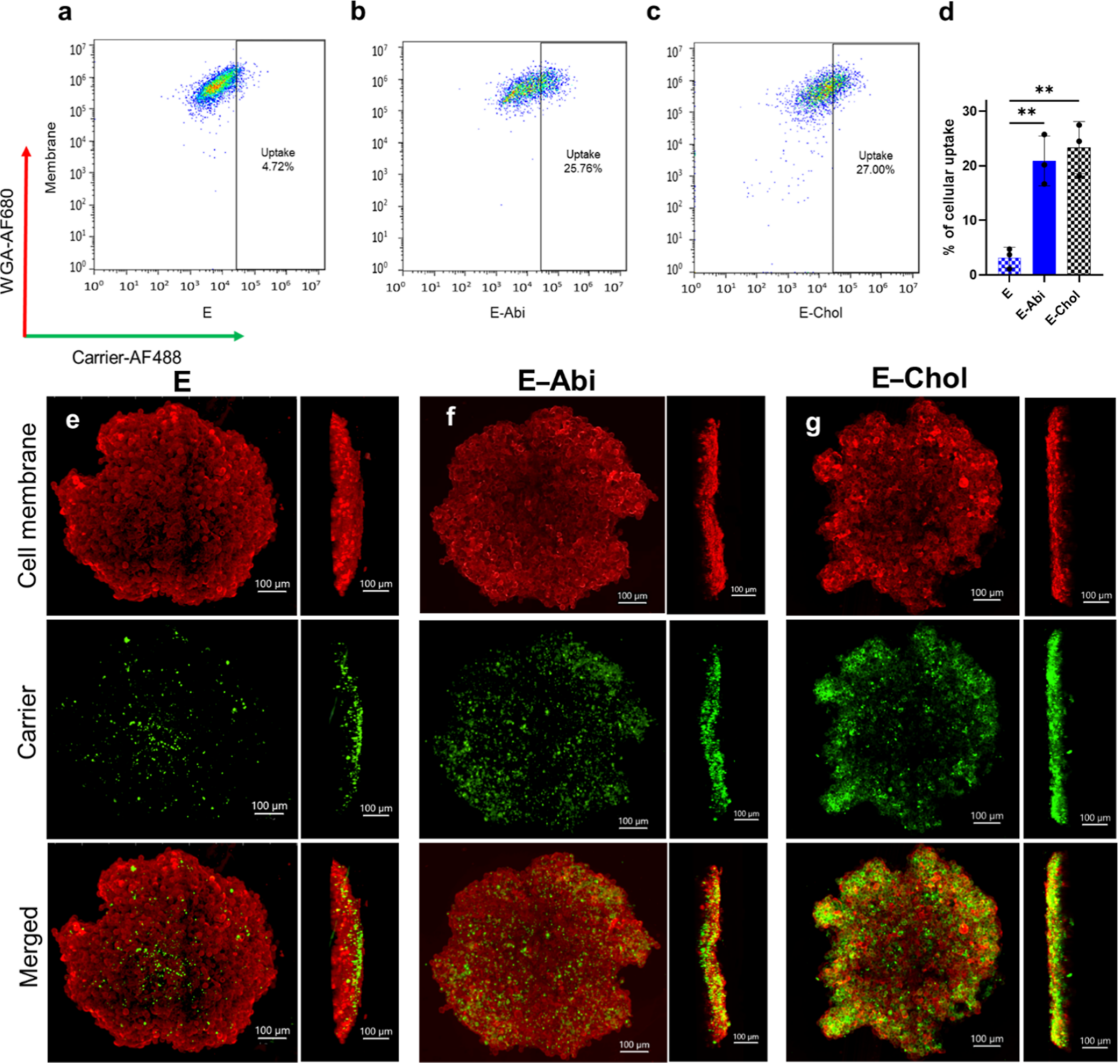
Lipidation-dependent modulation of biopolymer penetration in 3D tumor spheroids. (a–c) Representative bivariate flow cytometry dot plots of WGA-Alexa Fluor 680 (membrane label) versus carrier-Alexa Fluor 488. The gated population represents WGA^+^/Carrier^+^ cells, and the percentage of uptake-positive cells is indicated for each condition. DU145 spheroids were incubated overnight with labeled (a) E, (b) E–Abi, (c) E–Chol, dissociated to single cells, and analyzed by flow cytometry. (d) Bar graph summarizing the fraction of uptake-positive cells across treatments. (e–g) Confocal z-stack images of DU145 spheroids treated with E, E–Abi, or E–Chol for 24 h. WGA (AF680, red) marks the cell membrane, and constructs (AF488, green) indicate carrier localization. Data are mean ± s.d. (*n* = 3). Statistical significance was determined using one-way ANOVA with Tukey’s multiple comparisons test (**p < 0.01).

Finally, to demonstrate the generalizability of this ncPTM strategy, we extended this platform from abiraterone to galeterone (Gal), a second-generation CYP17A1 inhibitor with enhanced potency. The resulting conjugate (E–Gal) was synthesized with identical efficiency to E–Abi, supporting that plasticity of H-domain across structurally related sterols. In DU145 cells, E–Gal exhibited a faster and more potent response than E–Abi (reaching IC_50_ of 1.5 μM, 95% CI [1.2, 1.9] at 48 h; Figure S24), mirroring the higher potency of free Gal pharmacological profile of free Gal. Together, these results validate the platform’s modularity—the orthogonality between the H-domain and the carrier protein that enables the drug payload and the carrier sequence to be varied independently.

## Conclusion

In summary, these findings demonstrate that substrate plasticity of native lipidation machinery can be harnessed to create multifunctional protein–drug hybrids from pharmacologically active lipids. Using abiraterone and galeterone exemplars, we have shown that this strategy yields PDCs with significantly improved solubility and therapeutic performance compared to the small molecule drug. We envision leveraging the modularity of this platform to expand the chemical diversity of conjugated drugs and engineer the protein sequence to (i) improve pH-responsive release in the tumor microenvironment by optimizing the peptide interface to favor disassembly and accelerated hydrolysis under mildly acidic conditions, (ii) include targeting domains to enhance tumor accumulation beyond passive mechanisms, or (iii) drive nanoparticle formation for further pharmacokinetic modulation. Ultimately, these advances pave the way to evaluate the performance of these genetically programmable constructs in vivo.

## Experimental Section

### Materials

A complete list of materials and reagents is provided in the Supporting Information

### Computational Modeling

A model of the Hedgehog protein was generated using AlphaFold3,^65^ and the model exhibiting the highest degree of secondary structure was selected for subsequent simulations. To refine the predicted structure and sample conformational flexibility, molecular dynamics (MD) simulations were performed using NAMD3 ^66^ with the CHARMM36m force field.^67^ The simulation was run in a TIP3 water box. Initially, the protein backbone was restrained for 1 ns to relax side chains. This step was followed by equilibration of the entire system for 4 ns using a 1 fs time step. Subsequently, two independent equilibrations were performed for 200 ns per replica using a 2 fs time step. The MD simulation trajectories were used for principal component analysis (PCA) and k-means clustering with the MDAnalysis tool.^68^ The conformations were clustered into three groups. The average conformation of each cluster was selected and used as the receptor for docking. Cholesterol and abiraterone were docked independently into each representative Hedgehog conformation using AutoDock Vina^69^ via the PyRx interface.^70^ Docking calculations were performed with a grid box encompassing the *N*-terminal region. Representative binding poses were visualized and analyzed using VMD.

### Cloning

Plasmids encoding EH fusion constructs were generated as previously described.^13^ Briefly, the E-domain vector was digested with *AcuI* and *BglI*, while the H-domain vector was digested with *BglI* and *BseRI*. Gel-purified fragments were ligated and transformed into chemically competent *E. coli* EB5α cells. Correct assembly was confirmed by Sanger and Nanopore sequencing.

### Protein Expression and In-Lysate Modification

Proteins were expressed in *E. coli* BL21­(DE3) cultured in 2 × YT supplemented with kanamycin (60 μg mL^–1^) at 37 °C. Upon reaching an OD_600_ of 0.8, expression was induced using construct-specific conditions: (A,40) at 28 °C for 6 h with 0.1 mM IPTG, (V,30) and (V8/A2/K,80) at 16 °C for 14–16 h with 0.5 mM IPTG. Cells were harvested by centrifugation (4000 rpm, 30 min, 4 °C), resuspended in bTBS buffer (20 mM bis-Tris, 140 mM NaCl, pH 7.2), and sonicated on ice. Following clarification (14,000 rpm, 15 min, 4 °C), lysates were adjusted to contain 20 mM bis-Tris (pH 7.0), 140 mM NaCl, 5 mM TCEP·HCl, 5 mM EDTA, 1 mM Triton X-100 (included to enhance sterol solubility). Mixtures were equilibrated at room temperature for 10 min prior to the addition of sterol (1 mM final concentration, from a 20 mM stock in ethanol). Reactions were incubated at room temperature for 3 h. Control reactions were performed without Triton X-100 (Figure S5).

### Protein Purification

Sterol-modified ELPs were purified by leveraging their temperature and salt-responsive phase behavior. Following removal of unreacted sterol by centrifugation at 4 °C, NaCl was added to final concentrations of 2.5 M for (A,40) and 2.0 M for (V8/A2/K,80) and (V,30). Samples were incubated at 40 °C for 10 min to induce coacervation and centrifuged at 14,000 rpm for 15 min at 40 °C. Pellets were resuspended in 50% ethanol and lyophilized. Residual Triton X-100 was removed by ethanol trituration (3×). Final purification was achieved by preparative HPLC (>95% purity), and proteins were lyophilized prior to analysis.

### Fluorescent Labeling of Proteins

Primary amines were labeled by reacting proteins (50 μM) with Alexa Fluor 488 NHS ester (7.5 μM, targeting 15% labeling) and triethylamine (150 μM, 1.5 equiv relative to amines) in DMF (25 °C, overnight). Following the reaction, samples were diluted 10-fold with water, dialyzed against water, and then lyophilized. Unreacted dye were removed using Zeba Dye Removal Spin Columns according to the manufacturer’s protocol, followed by preparative HPLC for final purification. Labeling efficiency was determined spectrophotometrically (using the molar extinction coefficient of 73,000 M^–1^ cm^–1^). Conjugates were mixed with unlabeled protein to normalize the labeling density to 10% for biological studies.

### Sodium Dodecyl Sulfate Polyacrylamide Gel Electrophoresis (SDS–PAGE)

Proteins were resolved via SDS–PAGE on 4–20% Mini-PROTEIN TGX stain-free gels (200 V, 30 min) and imaged using a Gel Doc EZ system. The stain-free method selectively visualized the EH precursor and H domain via UV-induced tryptophan fluorescence (localized to the H domain). Purity was confirmed by subsequent staining with Coomassie Blue.

### Reverse-Phase High-Performance Liquid Chromatography (RP-HPLC)

Analytical RP–HPLC (Shimadzu LC-2030) was performed using a water/acetonitrile gradient of acetonitrile (0–90% over 45 min, with 0.1% trifluoroacetic acid in both mobile phases). Samples were filtered (0.2 μm PVDF) and injected (100 μL) at 1 mL min^–1^. Separation was achieved using Phenomenex Jupiter columns (5 μm, 300 Å, 250 × 4.6 mm). E–Chol was analyzed using a C4 column, while E–Abi, E–Gal, and E–Andro were analyzed on both C4 and C18 columns.

### Matrix-Assisted Laser Desorption/Ionization Time-of-Flight (MALDI-TOF)

MALDI–TOF MS spectra were acquired on a Bruker microflex LP (linear positive mode, 337 nm N_2_ laser). Protein samples (50 μM) were cocrystallized 1:1 (v/v) with matrix (sinapinic acid or α-cyano-4-hydroxycinnamic acid) dissolved in 70% acetonitrile containing 0.1% TFA. Following external calibration with cytochrome *c*, apomyoglobin, and aldolase, spectra were accumulated from 256 laser shots and processed using Bruker flexAnalysis software.

### Trypsin Digestion and LC–MS

Tryptic digestion (1:50 w/w, overnight, 37 °C) was performed to verify the identity and site-specificity of conjugation. After desalting with mixed-mode cation exchange StageTips, peptides were resolved by nano-LC (C18, mobile phase: water/acetonitrile with 0.1% formic acid, see Table S2 for gradient timing). Analysis was conducted on an Orbitrap Fusion Lumos mass via nano-ESI, utilizing MS1 and targeted MS2 acquisition modes. Orbitrap Lumos mass spectrometer via nanoelectrospray ionization (Table S2). Data were acquired using MS1 and targeted MS2 scans.

### Dynamic Light Scattering (DLS)

DLS measurements were performed on a NanoLab 3D system (LS Instruments, 90° scattering angle). Filtered samples (0.22 μm PVDF) were analyzed over a temperature ramp of 15–45 °C (1 °C steps, 2 min equilibration at each step). Correlation data were analyzed using LSLab software to determine the hydrodynamic radii (*R*_h_) and intensity-size distributions via the cumulant method and CORENN algorithm, respectively.

### VT-Turbidimetry

Temperature-induced phase separation was monitored by UV–Vis spectrophotometer (Cary 100) equipped with Peltier temperature control. Protein solutions (1–200 μM in PBS) were heated from 15–80 °C at 1 °C min^–1^ while monitoring the turbidity at 350 nm. The transition temperature (*T*_t_) was defined as the inflection point of the turbidity curve. Data were fitted to *T*_t_ = *T*_c_ + *m* × ln­(*C*), where *T*_c_ represents the reference transition temperature and m is the slope.

### Size-Exclusion Chromatography Coupled with Multiangle Light Scattering (SEC–MALS)

SEC–MALS analysis was conducted on a Waters chromatography system coupled to DAWN MALS and dRI detectors (Wyatt Technology). Samples (50 μL) were resolved on a Shodex Protein KW-804 column (8.0 × 300 mm) in PBS at a flow rate of 0.5 mL min^–1^. Absolute molar mass distributions were determined using ASTRA software (Wyatt Technology) assuming a standard protein refractive index increment (*dn/dc*) of 0.185 mL g^–1^.

### Drug Release Kinetics

Drug release from the E–Abi conjugate was evaluated under physiologically relevant conditions at different pH values and serum contents. Lyophilized protein samples were dissolved to a final concentration of 50 μM in (i) PBS (0% FBS), (ii) 10% FBS in PBS, or (iii) 50% FBS in PBS, each adjusted to pH 7.4 and pH 6.5. Samples were incubated at 37 °C. At each time point, aliquots were analyzed with RP-HPLC. Drug release was quantified by monitoring the appearance of the unmodified ELP peak corresponding to hydrolysis of the C-terminal ester linkage. The extent of abiraterone release was calculated using peak area integration according to

$$\%\;release=\frac{AUC\;of\;unmodified\;peak}{total\;AUC\;\left(unmodified+modified\right)}\times 100$$

Release kinetics were analyzed by nonlinear regression using a one-phase association model

$$y=\left(1-e^{-kt}\right)\times 100$$

where *k* represents the apparent first-order release rate constant. *t*_1_/_2_ was calculated as ln 2/*k*.

### Cell Culture

DU145 human prostate cancer cells (ATCC HTB-81) were cultured in high-glucose DMEM supplemented with 10% fetal bovine serum and 0.1% penicillin–streptomycin at 37 °C in a humidified 5% CO_2_ atmosphere. Cells were passaged at 80–90% confluency using 0.25% trypsin–EDTA and used between passages 5 and 30.

### Cell Viability Assay (2D and 3D)

For 2D, DU145 cells were seeded in 96-well plates at (5000 cells/well) in complete DMEM media overnight. Next day, the cells were treated with serial dilutions of conjugates (E–Abi, E–Chol & E–Gal) or free abiraterone (dissolved in DMSO) in serum-free DMEM, with untreated cells serving as controls. Control experiments confirmed that 0.05% DMSO (used for dissolving free drug) is nontoxic compared to untreated controls (Figure S25). Viability was assessed at 24, 48, 72, and 96 h post-treatment using MTT assay. At each time intervals, the media was replaced with 100 μL of MTT reagent (final concentration: 0.5 mg/mL). For E-Chol treatments, the protocol was slightly modified because initial runs showed cell dislodgement during complete medium removal (Figure S19a). To avoid this, ∼30 μL of medium was left in each well during the medium change, and the same handling was applied to matched controls. All the samples were incubated for 3 h, and the formazon crystals were solubilized in DMSO. The absorbance was measured at 570 nm (650 nm reference) using a SpectraMax i3 multimode plate reader (Molecular Devices, San Jose, CA, USA).

Growth inhibition was calculated according to

$$growth\;Inhibition\;\left(\%\right)=100\times \left(1-\frac{T_{t}-T_{0}}{C_{t}-C_{0}}\right)$$

where *T*_t_ represents the absorbance of drug-treated cells at time, C_t_ represents the absorbance of untreated control cells at the same time point, *T*_0_ represents the absorbance measured at the beginning of the experiment for treated wells, and *C*_0_ represents the absorbance of control wells at time zero. Absorbance values obtained from the MTT assay are proportional to the number of viable cells.

For 3D assays, DU145 spheroids were generated using the hanging-drop method by seeding 5 × 10^4^ cells per 20 μL drop and incubating for 48 h. Spheroids were transferred to an ultralow attachment 96-well plates. On day 3, the spheroids (10 per condition) were treated with 100 and 150 μM of E–Abi conjugates or free abiraterone. After 24 h, spheroids were enzymatically dissociated, and cell viability was quantified using MTT assay.

### Cellular Uptake

To evaluate internalization, DU145 cells were treated with E, E–Abi, and E–Chol (30 μM doped with 10% Alexa Fluor 488-labeled protein). Monolayers (2D) were incubated with labeled proteins in serum-free DMEM for 3 h, while spheroids (3D) were incubated for 24 h. Following fixation and counterstaining (WGA), samples were analyzed by confocal microscopy.

### Live/Dead Viability Assay

Live and dead cells were assessed using Calcein AM and propidium iodide (PI). Following treatment with conjugates and free drug, the cells were washed with PBS and incubated with Calcein AM/PI staining solution under light-protected conditions. 2D cultures were incubated with 1.5 μM Calcein AM/10 μM PI for 15 min, whereas Spheroids (3D) were incubated with 3 μM Calcein AM/10 μM PI for 30 min at 37 °C before fluorescence microscopy. Confocal microscopy (Zeiss LSM 980, Airyscan 2) was used to determine viability based on green (live) and red (dead) fluorescence signals.

### Confocal Fluorescence Microscopy

Confocal imaging was conducted using a Zeiss LSM 980 (Airyscan 2) for monolayers and Live/Dead assays, and an Andor Dragonfly 620 SR spinning-disk microscope for 3D spheroid uptake/penetration. Fluorophores (Alexa Fluor 488/680, Calcein AM, PI) were imaged using standard filter sets. Identical acquisition settings were maintained for comparative analysis. Data were processed using Fiji and Imaris software (Oxford Instruments).

### LC–MS Quantification of Intracellular Drug Release

Intracellular release of abiraterone from E–Abi conjugates was quantified by LC–MS/MS. DU145 cells (15000 cells/sample) were treated with free Abi and E–Abi, and lysed at the indicated time points (3, 24, 48, and 72 h) with 100 μL RIPA buffer. Clarified lysates were subjected to protein removal by adding 300 μL of cold acetonitrile to precipitate proteins. Matrix-matched calibration standards were prepared by spiking blank DU145 cell lysates with serial dilutions of free abiraterone. Analysis was performed on a Vanquish LC system coupled to a TSQ Quantis triple quadrupole mass spectrometer (Thermo Scientific). All samples and standards were diluted 1:10 using 20% acetonitrile containing 0.1% formic acid prior to analysis. Samples (5 μL) were injected onto a C18 column (2.1 × 30 mm, 3 μm C18, Uptisphere) and eluted with a gradient from 20 to 95% acetonitrile over 3.3 min. Electrospray ionization was performed at 4000 V with sheath gas set to 30 au, and with ion transfer tube and vaporizer set at 325 and 250 °C, respectively. Abiraterone was quantified by measuring the area under the curve of the top three product ions: *m*/*z* 156, 170 and 334.

### Flow Cytometry

Internalization of Alexa Fluor 488-labeled E, E–Abi, and E–Chol was quantified in DU145 monolayer and spheroid by flow cytometry. Cells were treated with proteins (30 μM, 3 h) in 2D monolayers and (30 μM, 24h) in 3D spheroids (3 spheroids per condition). These assays were performed with a lower protein concentration to avoid confounding uptake with cytotoxicity. Following incubation, cells were washed, dissociated/detached, and stained with WGA–Alexa Fluor 680. To distinguish internalized proteins from surface-bound signal, membrane-associated fluorescence was quenched by adding 0.02% Trypan Blue immediately prior to analysis. Uptake was reported as the percentage of Alexa Fluor 488-positive cells. Flow cytometry was performed on a BD FACSCelesta (BD Biosciences, USA) with 488 and 640 nm excitation lasers in medium-flow mode, and 20,000 events were recorded per sample. Data was analyzed using FlowJo v11 (BD Biosciences, USA).

### Uptake Inhibition Study

DU145 cells were seeded on a 96 well plate (6000 cells/well) and adapted overnight. The monolayers were pretreated with endocytosis inhibitors, dynasore, genistein, or cytochalasin D (final concentration; 20 μM) for 30 min. Internalization assays were carried out using Alexa Fluor 488-labeled E–Abi (30 μM doped with 10% Alexa Fluor 488-labeled protein)) for 3 h in the continued presence of inhibitors. Cells were washed, stained, and imaged by confocal microscopy as described in the 2D uptake assay.

## Supplementary Material



## Notes

Figures, tables, and additional experimental details (PDF)

## ∥

Miguel Guzman, Department of Biomedical Engineering, Northwestern University, Evanston, Illinois 60208, United States

## ⊥

Md Shahadat Hossain, Department of Pharmaceutical Technology, Faculty of Pharmacy, University of Dhaka, Dhaka 1000, Bangladesh.
