# Supplementary material for: Site-Specific Abiraterone Protein–Drug Conjugates via Hedgehog Autoprocessing
Source: ACS Appl Mater Interfaces. 2026 Apr 9;18(15):21488–99. doi: 10.1021/acsami.5c22490 (PMC13107367; doi:10.1021/acsami.5c22490)
Supplement: Supplementary file 1 [file am5c22490_si_001.pdf]

Supplementary Information

Site-Specific Abiraterone Protein–Drug Conjugates via Hedgehog Autoprocessing

Asma Gulzar,[1,§] Laiba Maryam,[1,§] Sisila Valappil,[1,§] Vivanthi Thanthrige,[1] Ebbing de Jong,[2] Miguel Guzman,[1†] Md Shahadat Hossain,[1‡] Atanu Acharya,[1,3] and Davoud Mozhdehi[1,3,*]

[1] Department of Chemistry, Syracuse University, 111 College Place, Syracuse, New York 13244, USA
[2] Upstate Medical University, Proteomics and Mass Spectrometry, Weiskotten Hall 4307 WHA, 766 Irving Avenue, Syracuse, New York 13210, USA
[3] BioInspired Syracuse: Institute for Material and Living Systems, Syracuse University, Syracuse, New York 13244, USA
* dmozhdeh@syr.edu
§ These authors contributed equally to this work.

Table of Contents

1. Materials S1
2. Supplementary Tables S2
3. Supplementary Figures S4
4. References........ S29

1. Materials

The chemically competent NEb5α, and BL21(DE3) cells, restriction enzymes, ligase, and corresponding buffers and DNA extraction kits were purchased from New England Biolabs (Ipswich, MA). Isopropyl b-D-1-thiogalactopyranoside (IPTG), apomyoglobin, cytochrome C, aldolase, sinapinic acid, and trifluoroacetic acid (TFA) were purchased from Sigma Aldrich (St. Louis, MO). Tryptone, yeast extract, sodium chloride, 2,2-Bis(hydroxymethyl)-2,2',2''-nitrilotriethano (Bis-Tris), tris-(2-carboxyethyl)phosphine, hydrochloride (TCEP), kanamycin, phosphate buffer saline (PBS), DMSO, acetonitrile, ethanol, cholesterol, abiraterone, ethylenediaminetetraacetic acid (EDTA), Dulbecco's Modified Eagle Medium (DMEM), trypsin–EDTA, and fetal bovine serum (FBS) were purchased from ThermoFisher Scientific (Waltham, MA). Galeterone and β-androstenol were obtained from Steroloids Inc. HPLC-grade acetonitrile was purchased from Fisher Scientific (Fair Lawn, NJ). MiniPROTEIN TGX stain free precast gels, precision plus protein unstained protein standards were purchased from Bio-Rad Laboratories, Inc. (Hercules, CA). Deionized water was obtained from Milli-Q system (Millipore SAS, France). DU145 (HTB-81) was obtained from the American Type Culture Collection (ATCC, Manassas, VA, USA). Oligonucleotides and gene fragments were purchased from Integrated DNA Technologies. Dynasore, genistein, and cytochalasin D were obtained from Enzo Life Sciences (Farmingdale, USA). All chemicals were used as received without further purification.

2. Supplementary Tables

Table S1. Sequences and molecular weights of proteins used in this study.

#	Name	Sequence	MW (Da)
1	**(A,40)**	*(M)*SKGPGAGVPGAGVPGAGVPGAGVPGAGVPGAGVPGAGVPGAGVPGAGVPGAGVPGAGVPGAGVPG<br>AGVPGAGVPGAGVPGAGVPGAGVPGAGVPGAGVPGAGVPGAGVPGAGVPGAGVPGAGVPGAGVPGAG<br>VPGAGVPGAGVPGAGVPGAGVPGAGVPGAGVPGAGVPGAGVPGAGVPGAGVPGAGVPGAGVPGAGVPGAGVP<br>GAGVPGKGGSGGSTVHG	16626.7
2	**(V,30)**	*(M)*GVGVPGVGVPGVGVPGVGVPGVGVPGVGVPGVGVPGVGVPGVGVPGVGVPGVGVPGVGVPGVGVP<br>GVGVPGVGVPGVGVPGVGVPGVGVPGVGVPGVGVPGVGVPGVGVPGVGVPGVGVPGVGVPGVGVPG<br>VGVPGVGVPGVGVPGVGVPGKGGSGGSTVHG	13284.6
3	**(V_8/A_2/K,80)**	*(M)*GVGVPGVGVPGAGVPGVGVPGVGVPGVGVPGVGVPGVGVPGAGVPGVGVPGVGVPGVGVPGGKGVGVPGVGV<br>PGAGVPGVGVPGVGVPGVGVPGVGVPGVGVPGAGVPGVGVPGVGVPGVGVPGGKGVGVPGVGVPGAGVPGVGVP<br>GVGVPGVGVPGVGVPGVGVPGAGVPGVGVPGVGVPGVGVPGGKGVGVPGVGVPGAGVPGVGVPGVGVPGVGVPGVGVPG<br>VGVPGAGVPGVGVPGVGVPGVGVPGGKGVGVPGVGVPGAGVPGVGVPGVGVPGVGVPGVGVPGVGVPGAGVPGV<br>GVPGVGVPGVGVPGGKGVGVPGVGVPGAGVPGVGVPGVGVPGVGVPGVGVPGVGVPGAGVPGVGVPGVGVPGVGVPGGK<br>GVGVPGVGVPGAGVPGVGVPGVGVPGVGVPGVGVPGVGVPGAGVPGVGVPGVGVPGVGVPGGKGVGVPGVGVPG<br>AGVPGVGVPGVGVPGVGVPGVGVPGVGVPGAGVPGVGVPGVGVPGVGVPGGKGGSGGSTVHG	35063.03
4	**Hedgehog (H)**	CFTPESTALLESGVRKPLGELSIGDRVLSMTANGQAVYSEVILFMDRNLEQMQNFVQLHTDGGAV<br>LTVTPAHLVSVWQPESQKLTFVFADRIEEKNQVLVRDVETGELRPQRVVKVGSVRSKGVVAPLTREG<br>TIVVNSVAASCYAVINSQSLAHWGLAPMRLLSTLEAWLPAKEQLHSSPKVVSSAQQQNGIHWYANAL<br>YKVKDYVLPQSWRHDGHHHHHHHHG	24917.6

Table S2. Nano-LC gradient program used for peptide separation

Time (min)	Flow (µl/min)	%B
0.00	0.500	3.0
5.00	0.350	5.0
40.00	0.350	85.0
45.00	0.350	85.0
46.00	0.350	3.0
50.00	0.350	3.0

Table S3. Theoretical and observed masses of trypsinized C-terminal fragments (GGSGGSTVHG-X).

X	Theoretical $[M+H]^+$ (Da)	Observed m/z
OH	814.8173	814.8179
Chol	1183.4523	1183.4602
Abi	1146.3023	1146.3025

Table S4. Fragment-ion assignments for the abiraterone-modified C-terminal tryptic fragment identified by LC–MS/MS.

Ion	Theoretical $[M+H]^+$ (Da)	Observed m/z
$[H]^+$	110.0718	110.0714
$[HG]^+$	213.0982	213.0982
$[HG\text{-}Abi]^{+2}$	272.6677	272.6678
$[Abi\text{-}OH]^+$	332.2328	332.2373
$[G\text{-}Abi]^+$	407.2693	407.2692
$[HG\text{-}Abi]^+$	544.3282	544.3274

3. Supplementary Figures

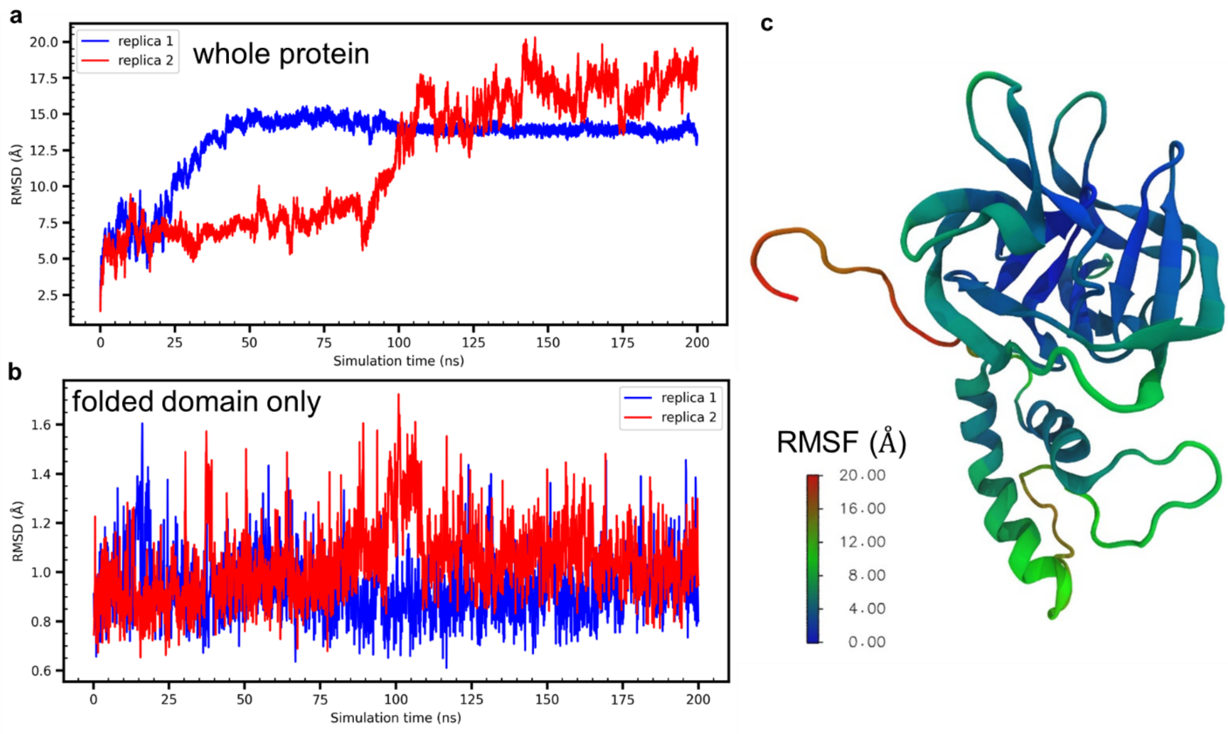

Figure S1. Molecular dynamics analysis of Hedgehog C-terminal domain flexibility. (a, b) The root-mean-square deviation (RMSD) along the simulation for the whole protein (a) and the folded domain only (b, residues 1 to 143) over 200 ns simulations, shown for two independent replicas. While the full-length construct exhibits larger RMSD values due to flexible terminal and loop regions, the folded domain remains structurally stable throughout the simulation with RMSD values near ~1 Å. **(c)** Root-mean-square fluctuation (RMSF) mapped onto the protein structure, highlighting higher flexibility in peripheral loops and terminal regions, with minimal fluctuations observed in the folded core. These results indicate a stable folded domain embedded within a dynamically flexible protein scaffold. See Table S1 for protein sequence.

Supplementary note. Because no experimental structure of hedgehog sterol recognition motif (SRM) is available and the initial AlphaFold3[1] model exhibited regions of low confidence, we used molecular dynamics (MD) simulations, and ligand docking to sample the accessible conformational landscape. Root-mean-square fluctuation (RMSF) analysis indicated that the dominant dynamics localize to the partially ordered SRM, which comprises two α-helices connected by flexible loops (Figure S1). Clustering of the MD trajectories identified three conformational states accessible at room temperature, distinguished primarily by the relative orientation between the folded catalytic core (intein) and the SRM (Figure S2). Although our simulations are conducted in the absence of a lipid bilayer, these ensembles are consistent with the proposed role of the SRM in membrane engagement and sterol presentation to the catalytic thioester intermediate.[2] Docking against representative structures from each cluster suggested that both Chol and Abi can adopt similar poses at the core–SRM interface (Figure 2b and Figure S3), with differences primarily localized to divergent interactions stabilizing the abi's pyridine ring. Given the presence of a nearby Asp, which could stabilize the pyridinium form and shift the local pKa of the abiraterone pyridine, the protonated state was used in the docking calculations

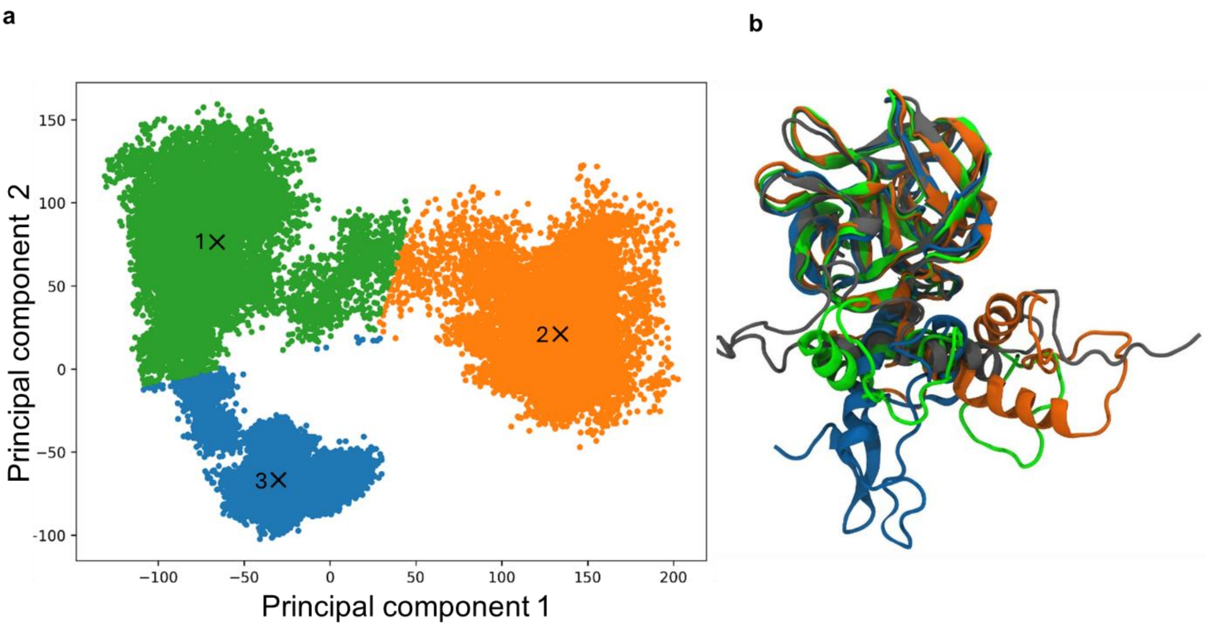

Figure S2. Comparison of clustered conformations with the initial equilibrated structure. (a) Two-dimensional projection of the conformational space showing distinct clusters identified from the MD trajectory; black crosses denote cluster centroids. **(b)** Structural superposition of representative conformations from each cluster aligned to the initial equilibrated structure (gray), highlighting overall conformational similarity and cluster-specific deviations.

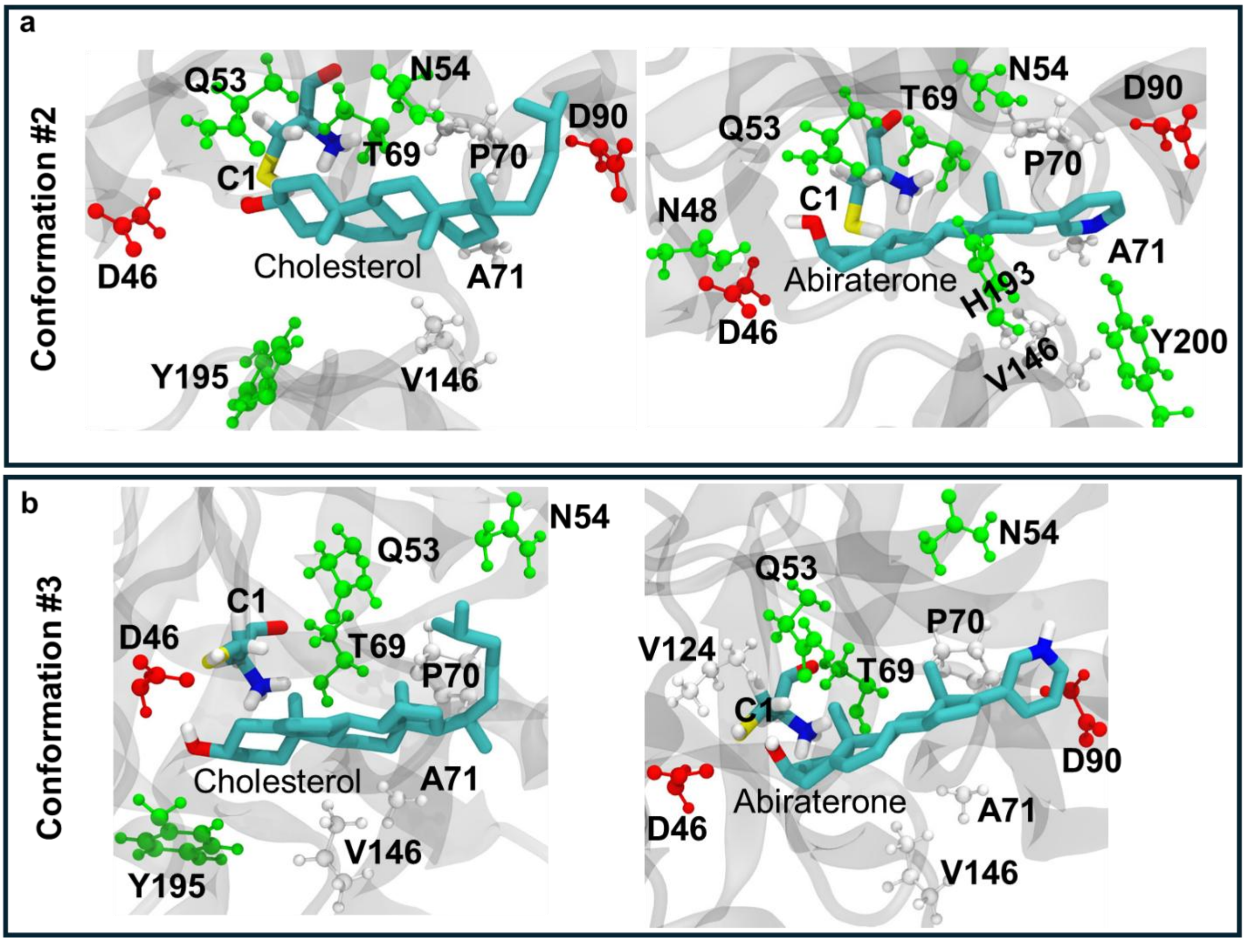

Figure S3. Comparative docking interactions in alternative conformational states. (a) Conformation 2 and **(b)** Conformation 3. Representative poses of cholesterol (left) and abiraterone (right) highlight key stabilizing interactions. Both ligands align their hydroxyl group with C1 and D46. Distinct interactions stabilize the sterol tails: hydrophobic residues P70 and A71 accommodate cholesterol, while Y200 and D90 engage the abiraterone pyridine ring, consistent with the structural plasticity of the active site formed at the interface of the core-SRM domain.

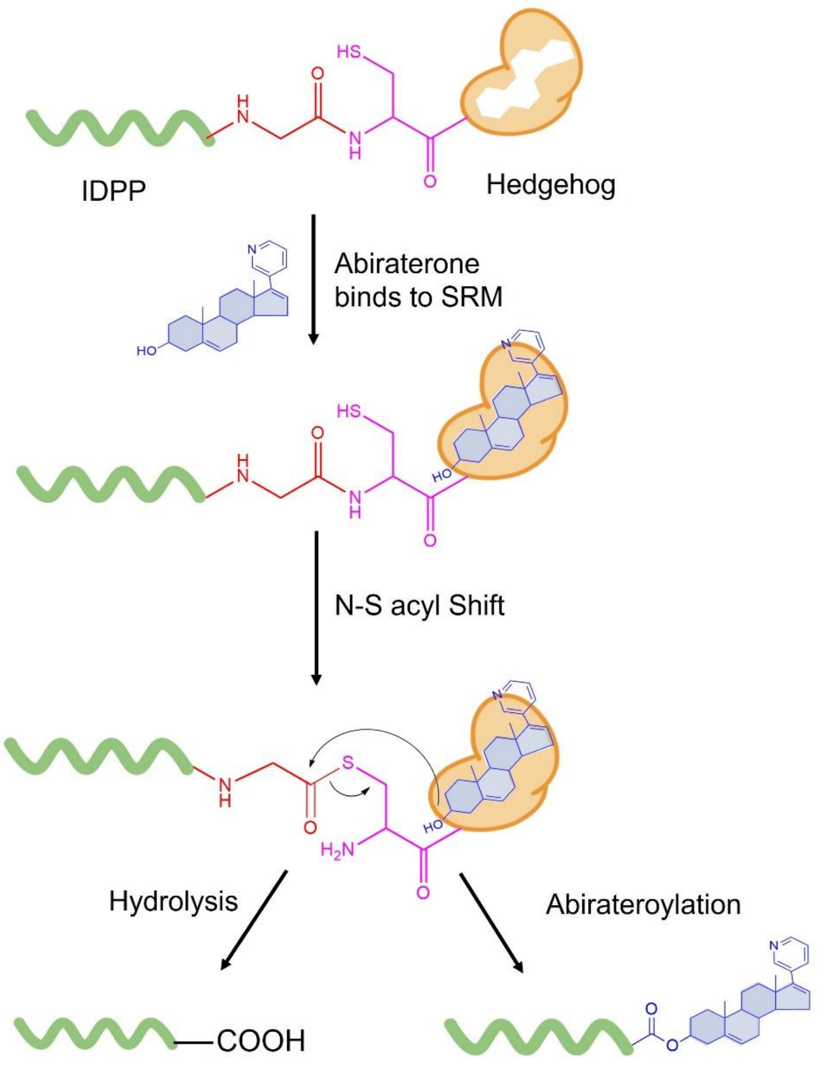

Figure S4. Mechanism of abiraterone-mediated autoprocessing of IDPP–hedgehog fusions. Binding of Abi to the sterol recognition motif (SRM), promotes N→S acyl shift, generating a reactive thioester intermediate at the junction of two domains. Nucleophilic attack by the hydroxyl group of Abi covalently links the drug to the protein C-terminus; while competing hydrolysis yields unmodified protein with a free carboxyl group.

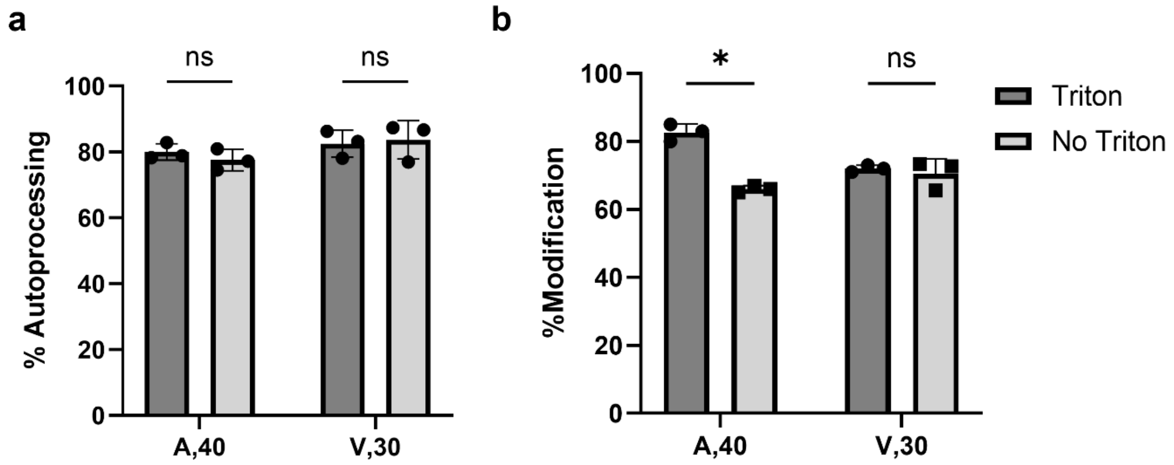

Figure S5. Effect of the nonionic surfactant Triton X-100 on hedgehog-mediated autoprocessing and abirateronylation. (a) Percent autoprocessing of representative constructs (selected for differing guest residue hydrophobicity) measured in the presence and absence of Triton X-100. **(b)** Percent sterol-modification of the same constructs under identical conditions. Data are presented as mean ± s.d. (n = 3), Statistical analysis was performed using an unpaired t-test (ns: not significant; * $p < 0.05$).

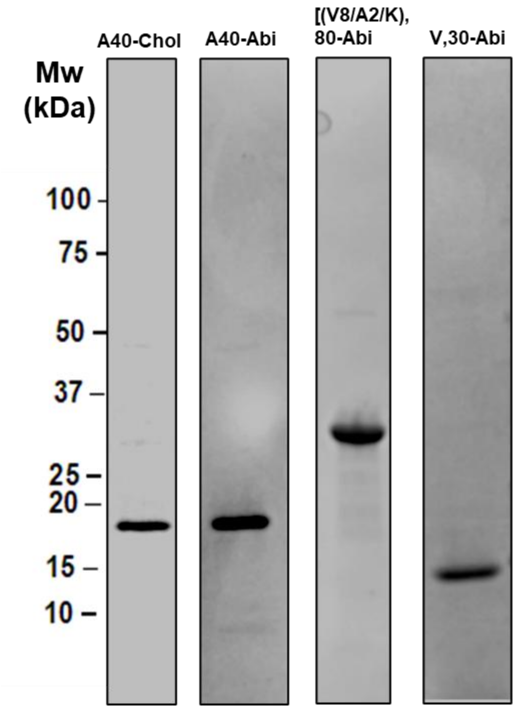

Figure S6. SDS–PAGE analysis of purified constructs. Coomassie Blue staining confirms the isolation of the constructs and the removal of starting materials and processed hedgehog domains.

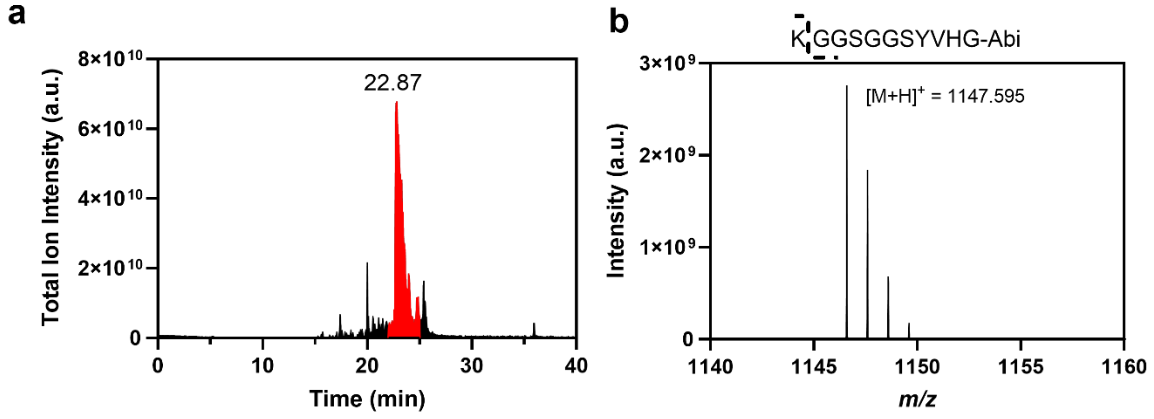

Figure S7. LC–MS analysis of trypsinized E–Abi protein. (a) Total ion chromatogram (TIC) and **(b)** high-resolution mass spectrum of the peptide corresponding to the highlighted peak. The identified fragment corresponds to the C-terminal peptide fragment bearing the abiraterone modification (GGSGGSVYHG–Abi).

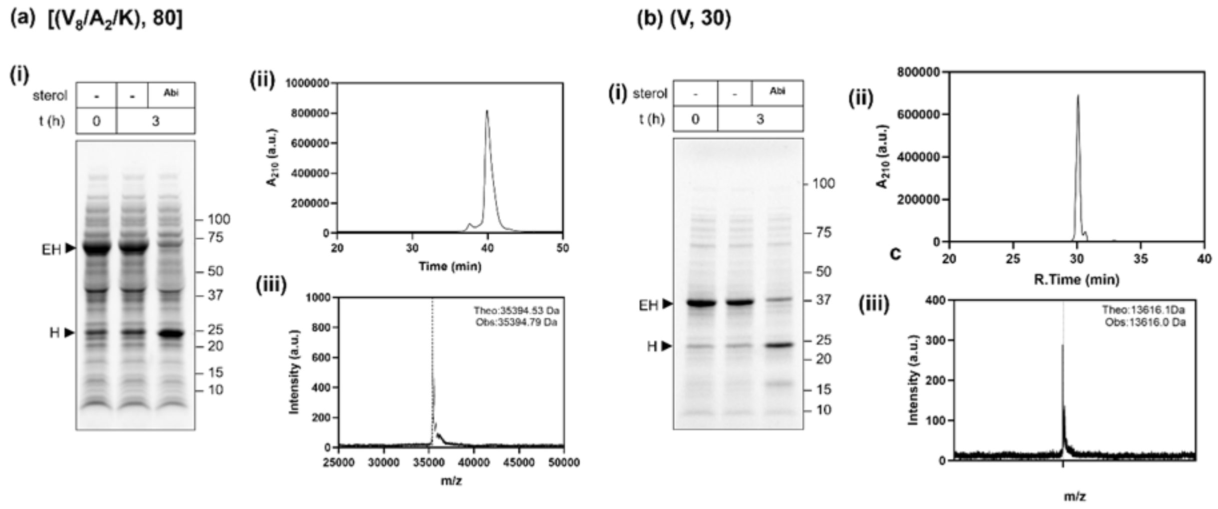

Figure S8. Hedgehog-mediated abirateronylation of model IDPPs derived from elastin scaffolds, $(GXGVP)_n$. (a) (V_8/A_2/K,80) and **(b)** (V, 30). Each panel shows: (i) Stain-free SDS–PAGE used to monitor the progress of EH precursor reaction with abiraterone, (ii) analytical RP–HPLC chromatogram, and (iii) MALDI–TOF–MS spectra of the conjugate.

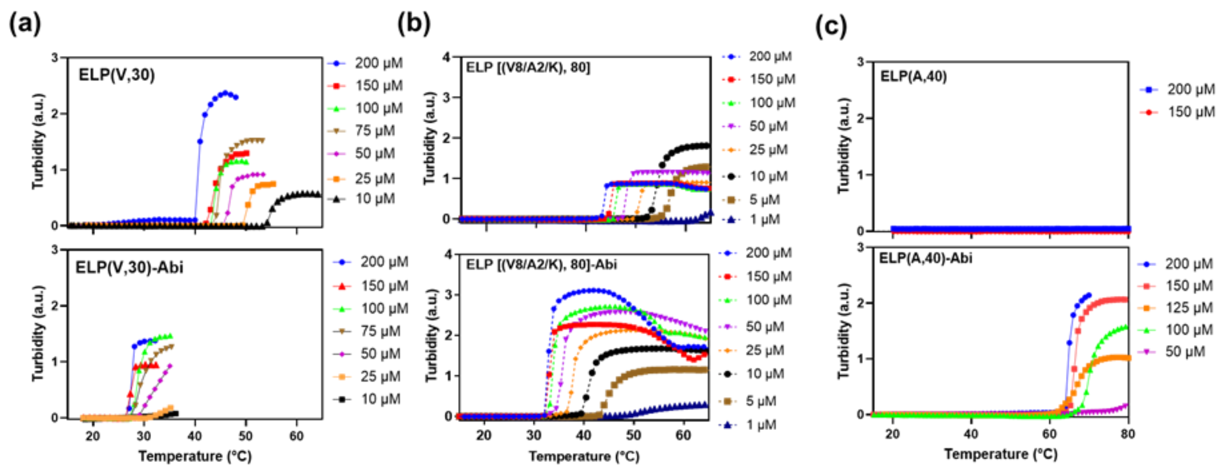

Figure S9. Representative turbidimetry profiles of model IDPPs before and after abirateronylation at various concentrations in PBS. (a) (V, 30), **(b)** (V_8/A_2/K,80, and **(c)** (A, 40) and their corresponding Abi conjugates (bottom panels). Unmodified (A, 40) shows no detectable phase transition within the accessible temperature range, even at the highest concentration tested. The data for unmodified (A, 40) in Figure 3b are derived from a previously developed model for *de novo* design of ELPs with Val and Ala as guest residues.[3]

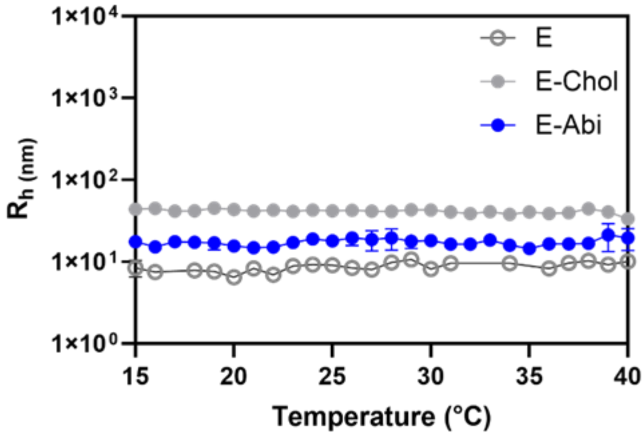

Figure S10. VT-DLS analysis of hydrophilic (A,40) isoforms. All three constructs exhibit a uniform hydrodynamic radius (R_h) across 15–40 °C, consistent with their colloidal stability over this temperature range.

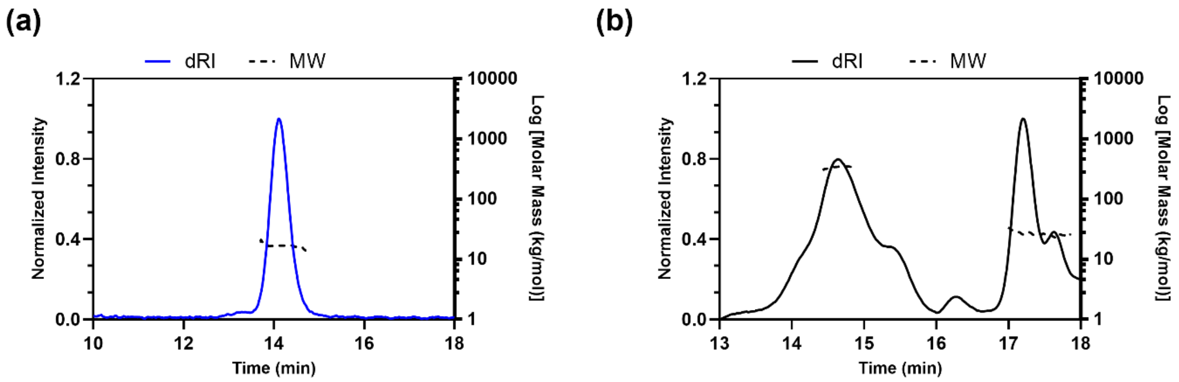

Figure S11. Size exclusion chromatography coupled with multi-angle light scattering (SEC-MALS) profiles of E–Abi and E–Chol. (a) E–Abi elutes as a single peak with a uniform molar mass profile (17 kDa), corresponding to a monomeric population. This indicates that E–Abi oligomers either dissociate upon dilution in the column or that the solution equilibrium heavily favors monomers. **(b)** E–Chol displays heterogeneous self-association, containing both high-molecular-weight aggregates (306 kDa, ~18-mers) and smaller species (24 kDa). Due to the high hydrophobicity of E–Chol, the late-eluting peaks may arise from nonspecific interactions with the column resin. This heterogeneity suggests that the hydrodynamic radius reported by DLS for E–Chol represents an average of a more complex equilibrium.

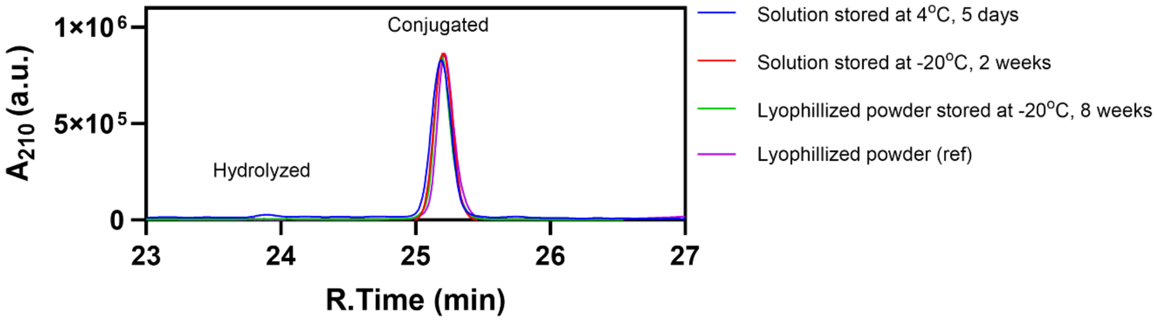

Figure S12. Storage stability of E–Abi. Analytical RP-HPLC chromatograms of samples stored as lyophilized powder at −20 °C (8 weeks), in frozen solution at −20 °C (2 weeks), or in solution at 4 °C (5 days). No significant changes in retention time or peak intensity were observed.

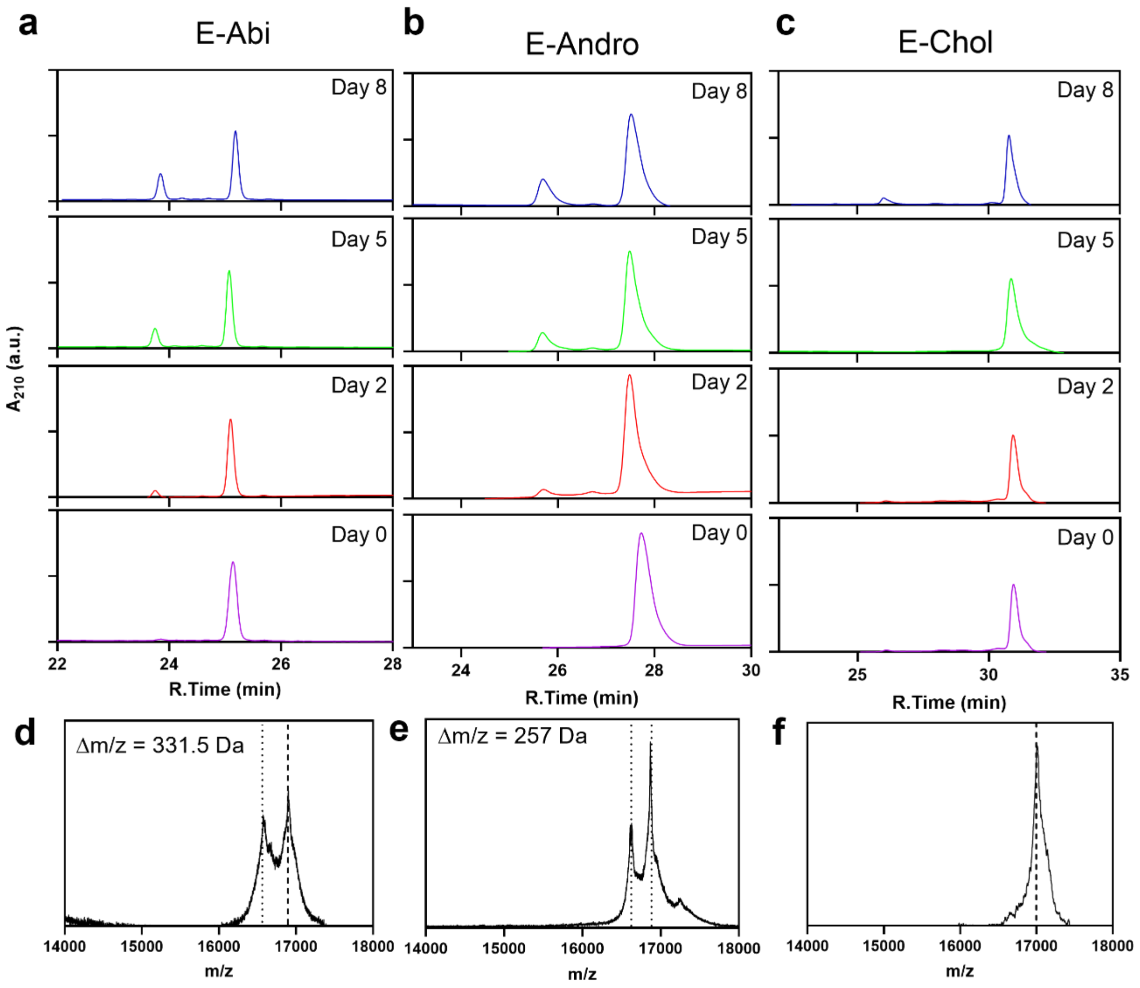

Figure S13. Monitoring sterol release via HPLC and MALDI-TOF. Representative analytical RP-HPLC of **(a)** E–Abi, **(b)** E–Andro, and **(c)** E–Chol incubated in PBS (pH 6.5, 37 °C) for 0–8 days. **(d–f)** MALDI-TOF spectra on day 8. **(d)** E–Abi displays separation by Dm/z = 331.5 Da, corresponding to hydrolysis of the C-terminal ester. **(e)** E–Andro shows separation by Dm/z = 257 Da, corresponding to androstanol loss via hydrolysis. **(f)** E–Chol shows a single peak, consistent with the intact conjugate.

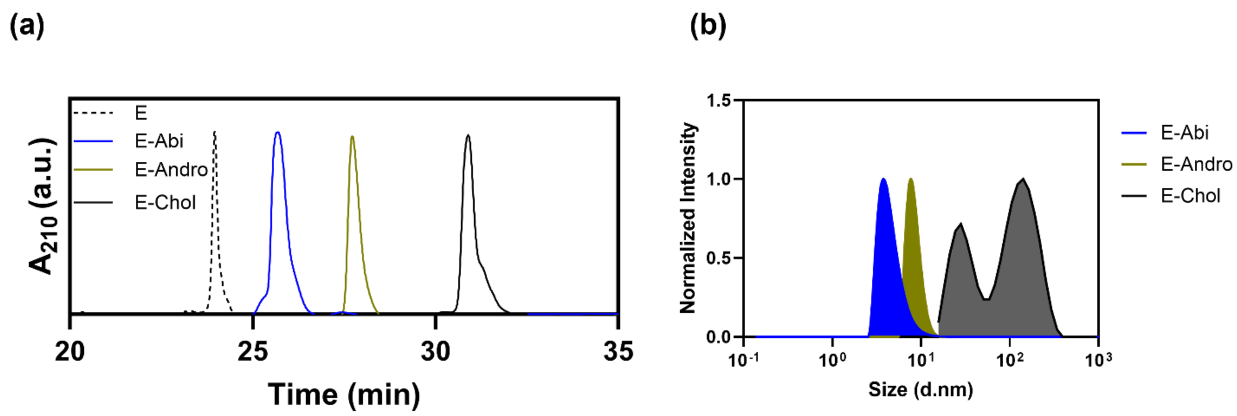

Figure S14. Correlation between sterol hydrophobicity and solution assembly. (a) RP-HPLC chromatograms of E (A,40) and corresponding sterolylated variants. sterol conjugates. Retention times confirm a hydrophobicity ranking of E–Abi < E–Andro < E–Chol under acidic mobile phase condition (0.1% TFA), which favors protonation of Abi. All constructs are analyzed using a C4 column, with a linear gradient of acetonitrile (0 to 90% over 45 min). **(b)** Intensity-size distributions show that assembly size scales with sterol hydrophobicity. The intensity-size distribution is consistent with the SEC-MALS data, indicating a favored monomeric population for E–Abi and a bimodal distribution for E–Chol. Cumulant analysis provides an intensity-weighted mean radius (R_h) for these populations. These assembly trends suggest a putative mechanism for the hydrolysis differences observed in Figure S13, wherein the dehydrated hydrophobic cores of E–Chol micelles potentially shield the ester linkage from water, whereas the smaller, more solvated assemblies of E–Abi and E–Andro allow greater water penetration and ester cleavage.

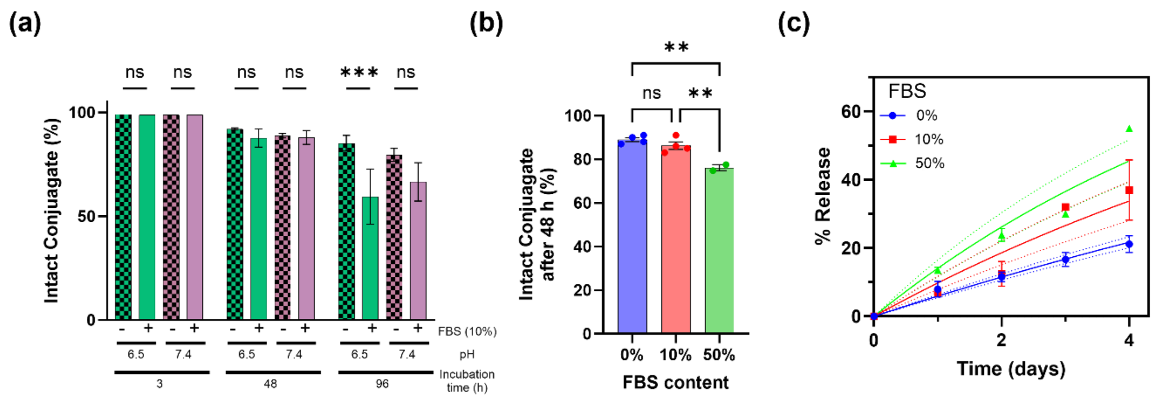

Figure S15. Stability and release kinetics of E–Abi in buffer and serum. (a) Effect of pH (6.5, 7.4) and FBS (0 vs. 10%) on conjugate stability quantified at 3, 48, and 96 h. Hydrolytic stability remains unaffected by tested pH but is accelerated by FBS at later time points. **(b)** Effect of serum concentration on E-Abi stability determined at 48 h endpoint. **(c)** Hydrolysis kinetics of E–Abi at different serum concentrations. Higher serum content accelerated hydrolysis. Data are mean ± s.d. (n = 3) for ELP (A,40)-Abi. Statistical significance calculated via two-way ANOVA (a) and one-way ANOVA (b) with Tukey's multiple comparisons test (ns, not significant; ** $p < 0.01$; *** $p < 0.001$). Dotted lines in c represent the 95% confidence intervals of the nonlinear regression fit to a first-order kinetics model.

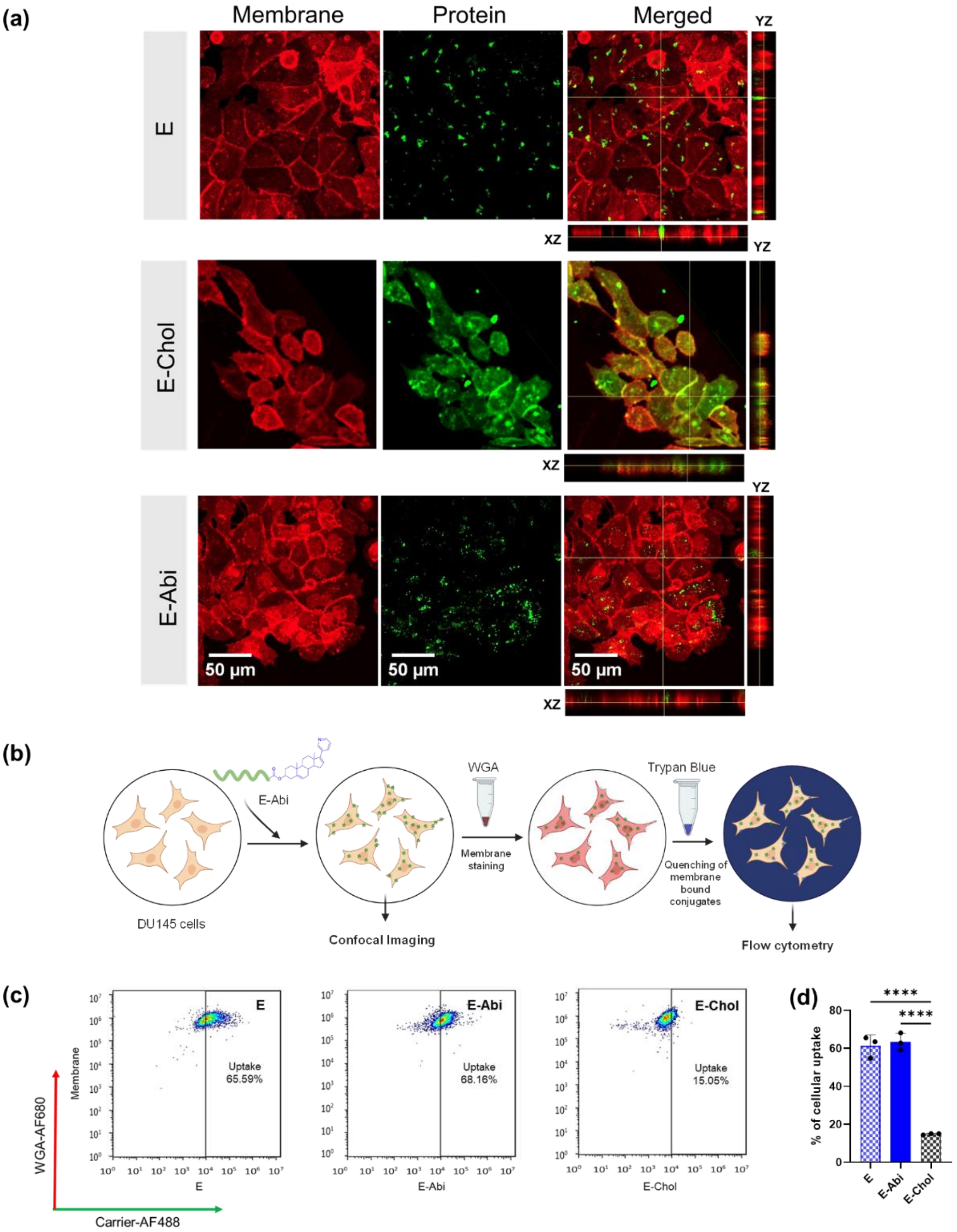

Figure S16. Lipidation-dependent modulation of biopolymer uptake in 2D DU145 monolayers. **(a)** Representative confocal images and orthogonal sections of DU145 cells treated with E, E–Chol, or E–Abi. E and E–Abi show punctate intracellular fluorescence, whereas E–Chol is predominantly membrane-associated. **(b)** Schematic illustration of the uptake workflow: DU145 cells were incubated with AlexaFluor 488-labelled carriers (green), counter stained with WGA (red) to visualize the cell membrane and treated with trypan blue prior to flow cytometry to quench membrane-bound fluorescence and quantify the cell population with internalized biopolymer conjugates. **(c)** Representative flow cytometry dot plots display cell populations with membrane WGA-Alexa Fluor 680 fluorescence versus Carrier-Alexa Fluor 488 fluorescence. The gated population represents $WGA^+/Carrier^+$ cells, and the percentage of uptake-positive cells is indicated for each condition. **(d)** Quantification of cellular uptake presented as mean ± s.d. (n = 3). Statistical significance was determined using ordinary one-way ANOVA with Tukey's multiple comparisons test (**** $p < 0.0001$).

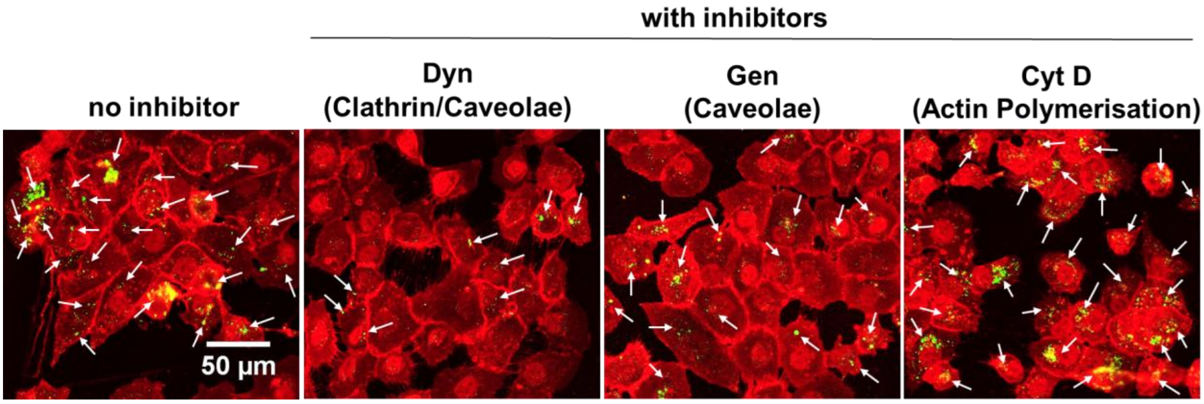

Figure S17. Intracellular uptake inhibition assay. Confocal fluorescence images of DU145 cells treated with fluorescently labeled E–Abi in the absence and presence of endocytosis inhibitors (20 µM, 30 min pre-incubation). Dynasore (Dyn) caused the strongest reduction in uptake, implicating dynamin-dependent pathways (clathrin- and/or caveolae-mediated endocytosis). Genistein (Gen) also significantly reduced uptake, supporting a caveolae-mediated mechanism. Cytochalasin D (Cyt D) showed minimal effect, indicating that actin-dependent macropinocytosis is not a primary pathway. Arrows indicate intracellular punctate signal in treated samples. Green: E–Abi (Alexa Fluor 488); Red: WGA membrane stain.

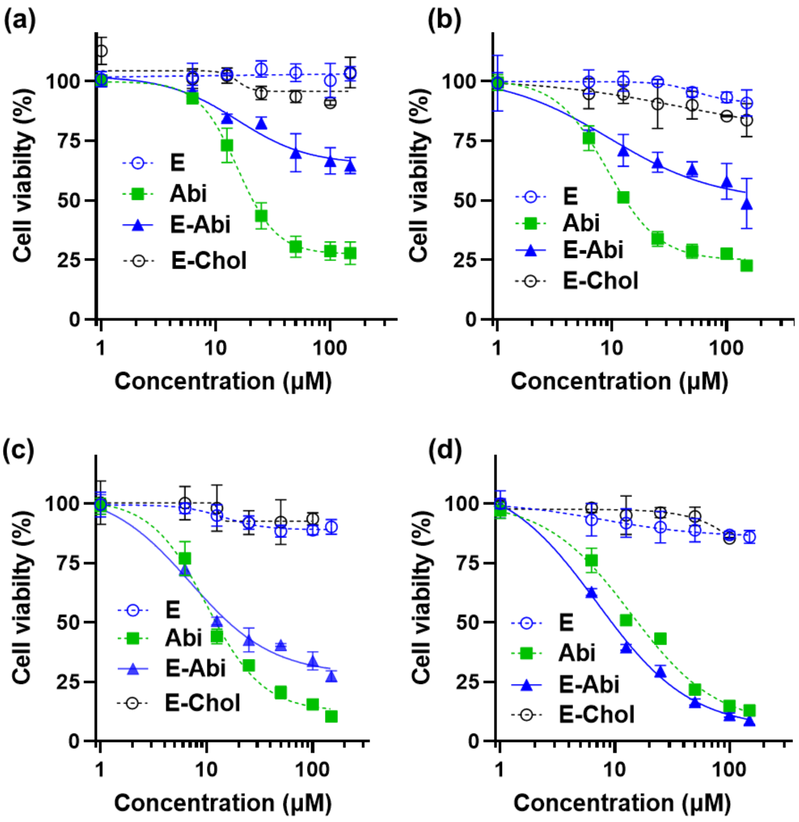

	IC_{50} (µM) [95% CI]			
	24 h	48 h	72 h	96 h
**Abi**	15 µM* [15-16]	14 µM* [12-16]	10 µM [9-11]	17 µM [16-18]
**E–Abi**	n.d.	n.d.	8 µM [7-9]	10 µM [9-11]

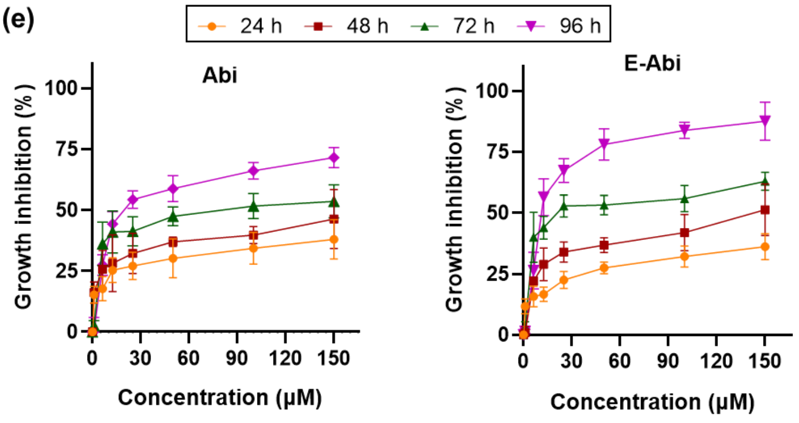

Figure S18. Time-dependent antiproliferative responses of DU145 cells in 2D monolayer culture. Dose–response curves for DU145 prostate cancer cells after **(a)** 24, **(b)** 48, **(c)** 72, and **(d)** 96 h exposure to E, free Abiraterone, E–Abi, and E-Chol. Viability was quantified with MTT assay, and the data were analyzed via a nonlinear dose–response fit. Data are shown as mean ± s.d. (n = 3). The IC_{50} values and their 95% confidence intervals are summarized in the table. n.d., not determined. Asterisks denote conditions where Abi did not reach a complete lower asymptote (~0% viability) within the tested concentration range. **(e)** Comparison of growth inhibition (%) across the same exposure times shows a stronger time-dependent increase for E–Abi compared to Abi, consistent with the proposed sustained hydrolysis of the pro-drug conjugate.

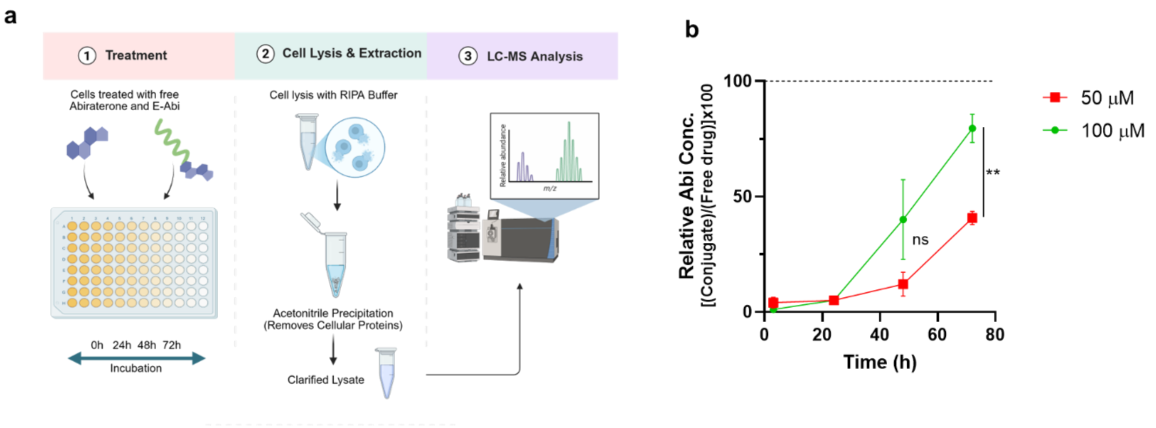

Figure S19. LC-MS quantification of time-dependent intracellular accumulation of abiraterone. (a) Mass spectrometry workflow for the quantification of intracellular Abi. **(b)** DU145 cells were treated with free Abi or E–Abi at 50 and 100 µM, and intracellular abiraterone levels were quantified at 0, 24, 48, and 72 h. Results are reported as the relative abiraterone concentration (conjugate/free drug), reflecting the effective amount of abiraterone released from E–Abi compared to free drug diffusing into the cell. The rapid uptake of the conjugate (Figure S16) paired with the gradual increase in relative drug concentration is consistent with the slow intracellular release of abiraterone from internalized conjugates. Data are mean ± s.e.m. ($n = 3$), Statistical significance was evaluated by an unpaired t-test (ns, not significant; ** $p < 0.01$).

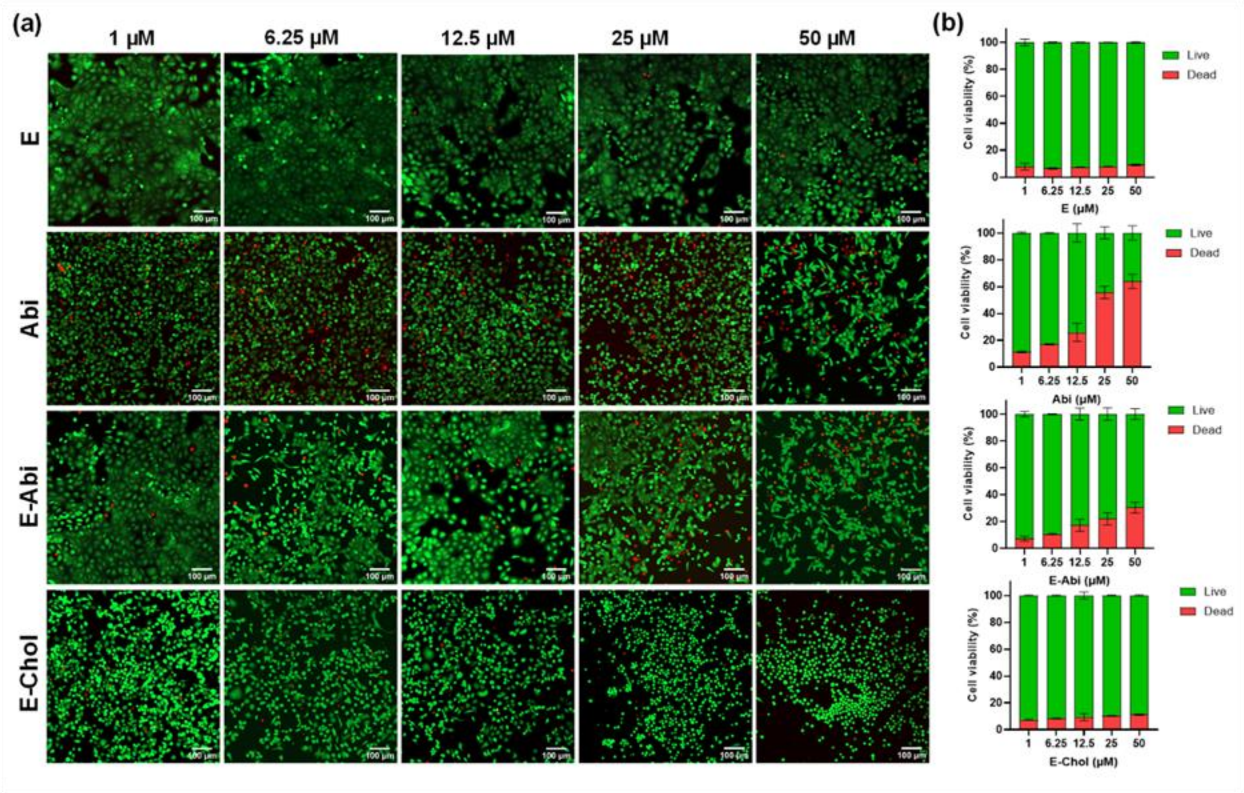

Figure S20. Live/dead imaging and quantification of DU145 cells after 24 h exposure to E, Abi, E–Abi, and E–Chol. (a) Representative calcein AM/propidium iodide micrographs (green = live, red = dead) across the indicated concentrations. **(b)** Image-based viability (%) for each condition; mean ± s.d. (n = 3). E and E–Chol show minimal cytotoxicity over the range tested, whereas Abi and E–Abi exhibit dose-dependent decreases in viability. We point out that E–Chol resulted in cell detachment at elevated concentrations (50 μM) in this loosely adherent cell line, likely arising due to its high amphiphilicity arising from the combination of a hydrophilic carrier with a strongly hydrophobic lipid.

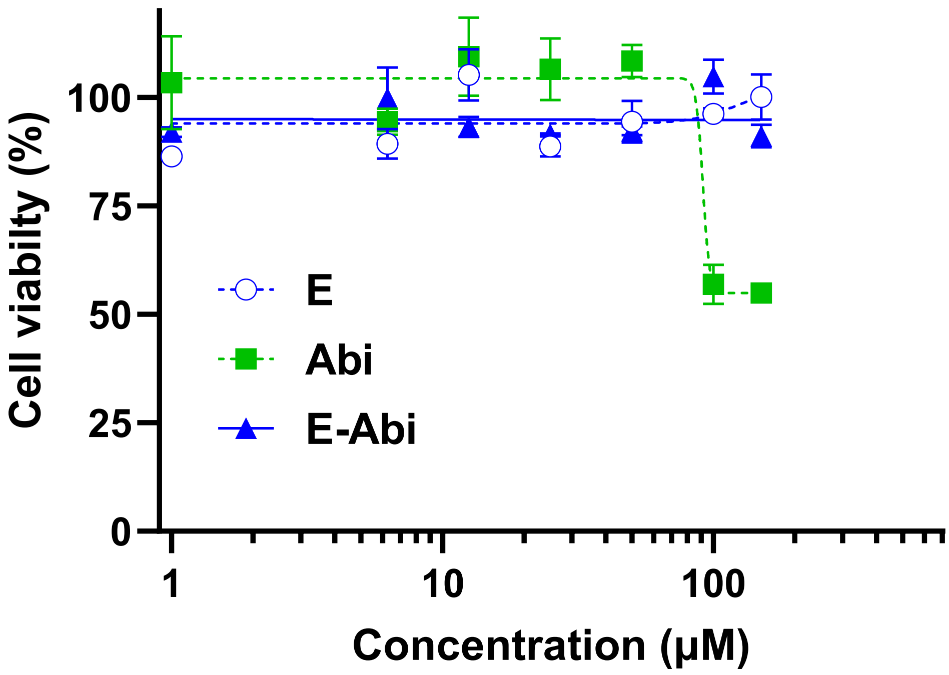

Figure S21. Cytotoxicity of abiraterone (Abi) and the protein–drug conjugate (E–Abi) in non-cancer cell models. Dose–response curves showing cell viability following treatment with increasing concentrations of Abi and E–Abi in NIH-3T3 fibroblast cells after 24 h treatment. Cell viability was determined by the MTT assay, normalized to untreated controls, and fitted using nonlinear regression (variable-slope, four-parameter logistic model). Data are mean ± s.d. (n = 3).

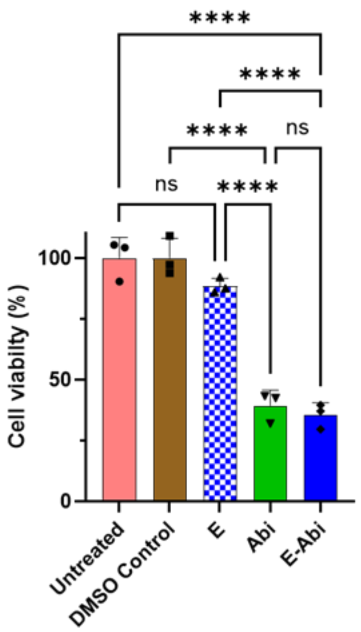

Figure S22. Viability of DU145 spheroids after 24 h treatment with 150 μM samples. Viability was assessed by the MTT assay for untreated control, DMSO control (used as a vehicle for Abiraterone), ELP (E), abiraterone (Abi), and the protein–drug conjugate (E–Abi). Data are mean ± s. d. (n = 3). Statistical significance was determined by one-way ANOVA with Sidak's multiple comparisons test (ns, not significant ; **** $p < 0.0001$).

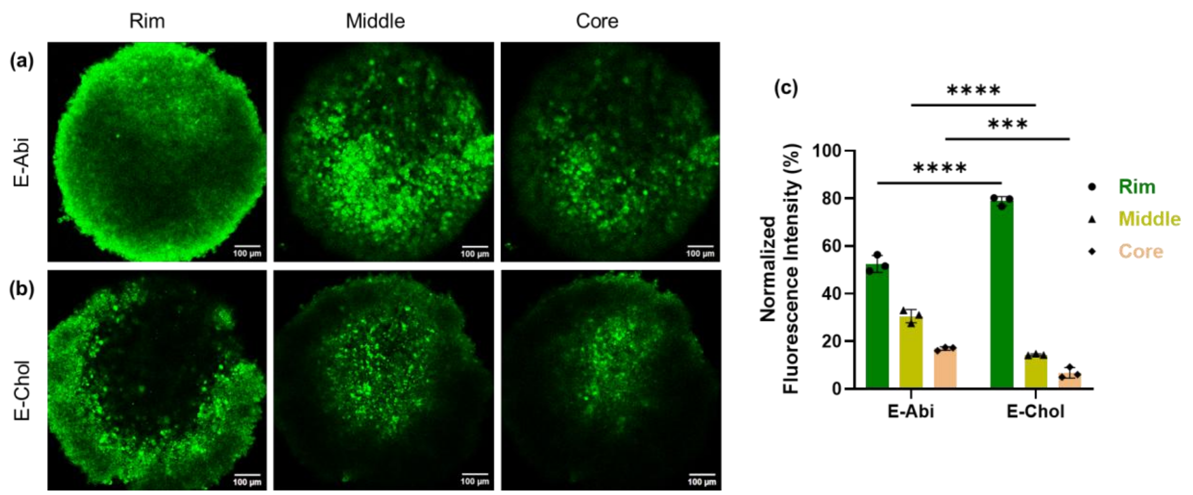

Figure S23. Analysis of PDC penetration depth in DU145 spheroids. DU145 tumor spheroids were incubated with AF488–labelled E–Abi or E–Chol (15 µM, 24 h), followed by confocal z-stack imaging and radial segmentation into rim, middle, and core regions. **(a,b)** Representative confocal z-stacks showing the distribution of (a) E–Abi and (b) E–Chol. Three slices were selected to represent depths of 0–120 µm (Rim), 120–240 µm (Middle), and 240–360 µm (Core). **(c)** Fluorescence distribution normalized to total spheroid signal. E–Chol remains trapped at the periphery, whereas E–Abi achieves significantly deeper penetration. Data are mean ± s.d. (n = 3). Statistical analysis was performed using a two-way ANOVA with Tukey's multiple comparisons test (*** $p < 0.001$, **** $p < 0.0001$).

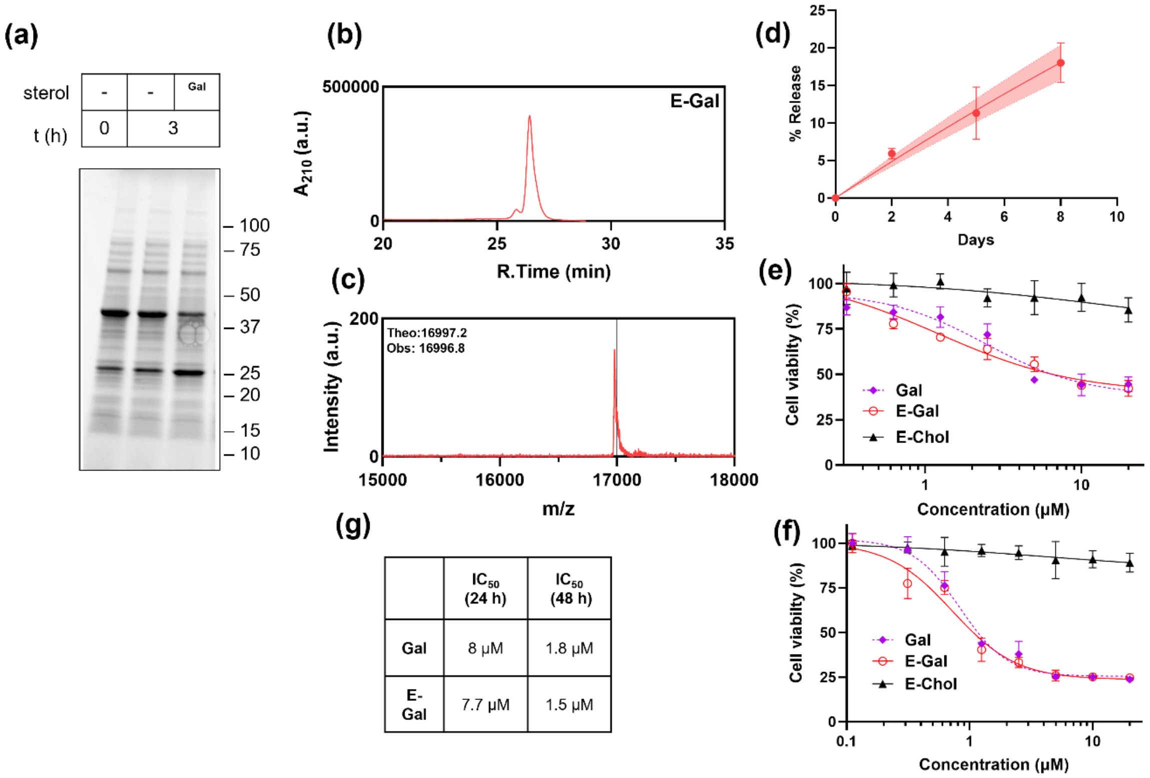

	IC_{50} (24 h)	IC_{50} (48 h)
Gal	8 µM	1.8 µM
E-Gal	7.7 µM	1.5 µM

Figure S24. Biocatalytic conjugation of galeterone and cytotoxicity of E–Gal in DU145 cells. (a) Stain-free SDS–PAGE used to monitor the progress of EH precursor reaction with galeterone. **(b)** Analytical RP–HPLC chromatogram, and **(c)** MALDI–TOF–MS spectra of the purified conjugate. **(d)** In vitro release kinetics of E-gal in PBS (pH 6.5, 37 °C). Shaded areas represent the 95% confidence intervals of the nonlinear regression fit to a first-order kinetics model. **(e,f)** Viability of DU145 cells after 24 h (e) and 48 h (f) exposure to E-Chol, free Gal, and E–Gal, quantified with MTT assay **(g)** Calculated IC_{50} values are summarized in the table. Data are mean ± s.d. (n = 3) fitted to a nonlinear dose–response curve.

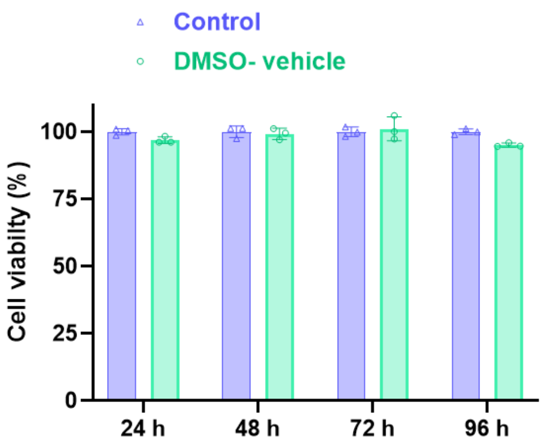

Figure S25. Effect of the DMSO vehicle toxicity. DU145 cell viability was quantified via MTT assay after incubation with 0.05% DMSO (vehicle) or media alone (untreated) for 24–96 h. No significant reduction in viability was observed in the vehicle group compared to controls, indicating that the DMSO concentration used for solubilizing Abi is non-toxic. Data are mean ± s.d. (n = 3).

4. References

(1) Abramson, J.; Adler, J.; Dunger, J.; Evans, R.; Green, T.; Pritzel, A.; Ronneberger, O.; Willmore, L.; Ballard, A. J.; Bambrick, J.; Bodenstein, S. W.; Evans, D. A.; Hung, C.-C.; O'Neill, M.; Reiman, D.; Tunyasuvunakool, K.; Wu, Z.; Žemgulytė, A.; Arvaniti, E.; Beattie, C.; Bertolli, O.; Bridgland, A.; Cherepanov, A.; Congreve, M.; Cowen-Rivers, A. I.; Cowie, A.; Figurnov, M.; Fuchs, F. B.; Gladman, H.; Jain, R.; Khan, Y. A.; Low, C. M. R.; Perlin, K.; Potapenko, A.; Savy, P.; Singh, S.; Stecula, A.; Thillaisundaram, A.; Tong, C.; Yakneen, S.; Zhong, E. D.; Zielinski, M.; Žídek, A.; Bapst, V.; Kohli, P.; Jaderberg, M.; Hassabis, D.; Jumper, J. M. Addendum: Accurate Structure Prediction of Biomolecular Interactions with AlphaFold 3. *Nature* **2024**, *636* (8042), E4–E4. https://doi.org/10.1038/s41586-024-08416-7.

(2) Purohit, R.; Peng, D. S.; Vielmas, E.; Ondrus, A. E. Dual Roles of the Sterol Recognition Region in Hedgehog Protein Modification. *Commun. Biol.* **2020**, *3* (1), 250. https://doi.org/10.1038/s42003-020-0977-2.

(3) McDaniel, J. R.; Radford, D. C.; Chilkoti, A. A Unified Model for De Novo Design of Elastin-like Polypeptides with Tunable Inverse Transition Temperatures. *Biomacromolecules* **2013**, *14* (8), 2866–2872. https://doi.org/10.1021/bm4007166.
